# Afforestation‐Related Fertilisation Quickly Turns Barren Cutaway Peatland Into a Carbon Dioxide Sink

**DOI:** 10.1111/gcb.70644

**Published:** 2025-12-17

**Authors:** Alexander J. V. Buzacott, Kari Laasasenaho, Risto Lauhanen, Kari Minkkinen, Paavo Ojanen, Gopal Adhikari, Liisa Jokelainen, Lassi Päkkilä, Hannu Marttila, Annalea Lohila

**Affiliations:** ^1^ Institute for Atmospheric and Earth System Research/Physics, University of Helsinki Helsinki Finland; ^2^ Seinäjoki University of Applied Sciences Seinäjoki Finland; ^3^ Department of Forest Sciences University of Helsinki Helsinki Finland; ^4^ Natural Resources Institute Finland Helsinki Finland; ^5^ Water, Energy and Environmental Engineering Research Unit, Faculty of Technology University of Oulu Oulu Finland; ^6^ Climate System Research, Finnish Meteorological Institute Helsinki Finland

**Keywords:** CH_4_, eddy covariance, land use change, N_2_O, NEE, peat extraction, radiative forcing, surface albedo

## Abstract

Energy peat extraction has declined rapidly in Europe in recent years, leaving thousands of hectares of land requiring after‐use management and planning. A popular after‐use option, afforestation, is understudied and there is a limited understanding of its overall effect on greenhouse gas (GHG) and energy exchange. In this study, we present a multi‐year record of eddy covariance fluxes of carbon dioxide (CO_2_), energy fluxes and surface albedo, chamber measurements of methane (CH_4_) and (N_2_O), and estimates of lateral carbon (C) losses from dissolved organic carbon (DOC) measurements from a cutaway peatland in Finland during the first 3 years of afforestation. The site was fertilised with wood ash and 2‐year‐old Scots pine ( *Pinus sylvestris* ) saplings were planted. Wild vegetation emerged at the site in the first summer after fertilisation. Satellite‐derived leaf area index data showed clear year‐on‐year increases, and there was good agreement with changes in CO_2_ fluxes over the full study period. The albedo of the site increased with plant cover annually, resulting in negative radiative forcings and net CO_2_‐equivalent (CO_2_‐eq) removals. After being a source of CO_2_ in the first year (144 ± 20 g CO_2_‐C m^−2^ year^−1^), the site transitioned to a sink for the next two study years (−36 ± 12 and −19 ± 19 g CO_2_‐C m^−2^ year^−1^). Annual fluxes of CH_4_ and N_2_O were small but not negligible. Net annual C losses stopped after 1 year, where DOC losses offset CO_2_ uptake, and the afforestation resulted in a mean change in the annual net ecosystem C balance of 172 g C m^−2^ year^−1^ and radiative balance of −688 g CO_2_‐eq m^−2^ year^−1^ over a 100‐year time horizon. Cutaway peatlands typically remain long‐term sources of C if abandoned, and our results indicate that the afforestation process can rapidly revegetate barren peatlands and halt net C losses.

## Graphical abstract

Afforestation is the most popular after‐use option of former peat extraction areas in Finland, but it is not well known if afforestation of these lands is climate‐friendly. We measured the exchange of greenhouse gases and estimated aquatic carbon losses to understand the net climate impact of a former peat extraction site in Finland. Our data showed that afforestation‐related fertilisation of the land spurred the emergence of wild vegetation on formerly bare peat soil and net carbon losses were quickly halted.

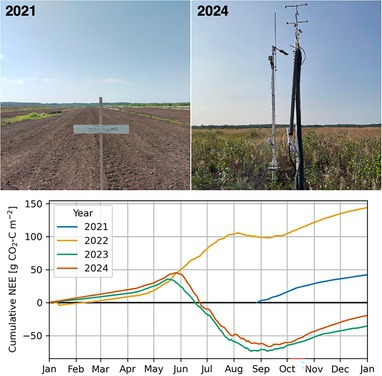

## 1 Introduction

Utilisation of peatlands for agriculture, forestry, or peat extraction requires enhanced drainage, which disrupts the natural carbon (C) accumulation of peatlands and typically turns them into sources of C. The extraction of peat, most commonly for energy or horticultural purposes, results in high emissions of greenhouse gases (GHG) and a rapid loss of C stocks (Wilson et al. 2015). Most of the leading peat extractors are in Europe, primarily Finland, Ireland, and Germany, where rates of energy peat extraction have recently declined because of changes in the European Union (EU) emission trading market, which has resulted in energy peat extraction becoming economically unattractive (Räsänen et al. 2023). The sudden decrease in extraction has left tens of thousands of hectares of land requiring after‐use management and planning. Former peat extraction areas that are abandoned can be persistent sources of carbon dioxide (CO_2_) emissions because the ditches installed to facilitate extraction are often not blocked or filled afterwards, leaving the peat drained and exposed to oxygen, where it can undergo microbial decomposition (Maljanen et al. 2010; Swenson et al. 2019; Tuittila et al. 1999; Waddington et al. 2002). Furthermore, functional peatland vegetation may not grow for some time because of harsh hydrological and soil conditions (Price and Whitehead 2001; Taylor and Price 2015; Van Seters and Price 2002). Abandoned sites may become CO_2_ sinks after several years or decades in the right conditions where there is recovery of the water table, soil hydrology, and re‐establishment of peatland vegetation (Samaritani et al. 2011; Wilson et al. 2013; Yli‐Petäys et al. 2007).

Peat extraction for horticulture typically cuts the uppermost layer of peat where there is *Sphagnum* (peat moss) peat, and extracted peatlands are termed cutover peatlands. Since only a thin layer can be removed, the area undergoing peat extraction tends to be large relative to the amount of peat harvested. In Canada, where peat has been predominantly extracted for horticulture, approximately 35,000 ha of land have undergone extraction (Harris et al. 2022) and the typical annual peat production has been approximately 1.3 million tonnes (USGS 2023). Peat extraction for energy removes a thicker layer of peat, and the remaining residual layer tends to be thin, and hence they are termed cutaway peatlands. On an annual mass basis, 50%–70% of peat was extracted for energy compared to 20%–35% for horticulture globally over the period 2002–2021 (USGS 2023). Finland was the global leader for peat extraction and was responsible for around 25% of the annual share by mass between 2001 and 2021, with an average production of 8.1 million tons per year (USGS 2023). As of 2019, the area under active peat extraction in Finland was estimated to be 56,000 ha (Laasasenaho et al. 2023), and the best estimate of historical extraction is that up to 114,000 ha have been used (GTK 2020). Peat extraction has steadily declined in Finland, and the share of peat energy in the electricity market has decreased from a maximum of 8.3% in 1996 to 1.1% in 2024 (Statistics Finland 2025). In addition to changes in the EU emission trading market, peat extraction has declined in Finland because of changes in national GHG emission policy and energy taxation (Laasasenaho et al. 2022).

The after‐use options of former peat extraction areas depend on the laws in each country and the type of extraction. For example, in Canada, where peat has been extracted almost only for horticultural use, active restoration is preferred (Räsänen et al. 2023) and the most common restoration technique is transplanting natural peat moss, which is viable on cutover peatlands (Graf and Rochefort 2016; Nugent et al. 2018). In Finland, peat extraction areas are mostly privately owned, and the landowners may freely choose what to do with their land after extraction has ceased. The most popular after‐use option in Finland is afforestation by a clear margin, where it was chosen by 71% of participants in a national survey (Laasasenaho et al. 2023). In the Baltic countries (Estonia, Latvia, and Lithuania), most peat extraction occurred during the Soviet occupation period, and extracted lands were mostly abandoned after the collapse in the early 1990s; however, afforestation was also a common after‐use (Karofeld et al. 2017). Afforestation of cutaways is also of interest in Ireland (Renou‐Wilson et al. 2008, 2010; Renou‐Wilson and Byrne 2015) and Sweden (Zetterberg et al. 2004).

Afforestation is the practice of establishing a forest where there has been no forest previously or where there was no forest for at least several decades. On peat extraction areas, afforestation transitions the land cover from bare and dark peat soil to vegetated and large changes in the exchange of GHGs and energy can be expected. When afforesting peat extraction areas, fertiliser is applied since the residual peat is poor in mineral nutrients such as phosphorus, potassium and boron, but usually rich in nitrogen (N) (Kaunisto 1997; Laiho and Laine 1994; Moilanen et al. 2015). Tree stands are established either by planting saplings or by sowing. The initial application of fertiliser can lead to rapid growth of ground vegetation (Huotari et al. 2007), which can outpace C accumulation in the small tree saplings and may become an important C stock (Huotari et al. 2009). However, the short‐ and long‐term net climate impact of afforesting these sites remains uncertain, as there are limited published studies providing continuous data on GHG exchange from afforested peat extraction sites. Previous research has used manual chamber observations (Mäkiranta et al. 2007; Soini et al. 2010), productivity estimates from trenched plots (Jauhiainen et al. 2023), or a combination of productivity and chamber data (Bravo et al. 2018), which typically result in high uncertainty of annual GHG exchange. It is therefore unclear what the dynamics of GHG exchange are during the afforestation process and, for example, whether CO_2_ uptake by plants can surpass CO_2_ emissions from peat decomposition.

In this study, we present a more than 3‐year record of eddy covariance (EC) measurements, chamber measurements, and lateral dissolved organic carbon (DOC) loss estimates from a cutaway peatland site in Finland undergoing afforestation. We investigate the covariation of GHG and energy exchange with land cover development to understand the effect of afforestation. The aims of the study were to determine the effect of afforestation and related management on the: (1) land cover of the site, (2) GHG and energy flux dynamics and partitioning, and (3) net annual C and radiative balances to assess the net climate impact.

## 2 Materials and Methods

### 2.1 Study Site

**FIGURE 1:**
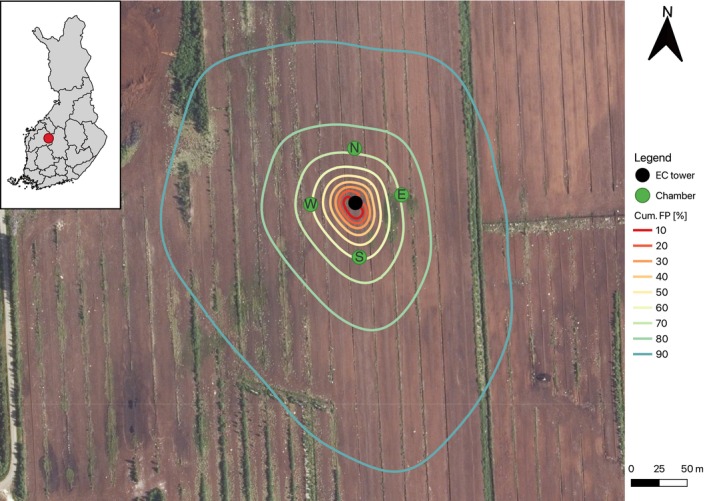
Aerial overview of the study site in 2021. Chamber sampling points north (N), east (E), south (S) and west (W) were spaced approximately 50 m from the eddy covariance (EC) tower. The cumulative footprint (Cum. FP) contours were derived from the full study period eddy covariance data using the Kljun et al. (2015) footprint climatology model. The inset displays the location of the study site within Finland. Map lines delineate study areas and do not necessarily depict national boundaries.

Measurements were conducted at Naarasneva (62.86390° N, 24.08382° E, Elevation: 179 m), a cutaway peatland located in Soini municipality, South Ostrobothnia, Finland (Figure 1). The mean annual temperature from 1990 to 2024 is 3.6°C ± 0.8°C, and the mean annual precipitation is 633 ± 90 mm (extracted from Finnish Meteorological Institute [FMI] gridded data; Aalto et al. 2016). Before disturbance, Naarasneva was an aapa mire and the measurement site was a mostly treeless and at places sparsely treed minerotrophic fen. Preparation for peat extraction began in 1974, and harvesting commenced in 1980 (Ruususaari et al. 2018). During preparation, ditches were installed approximately at a 20 m interval, and the depth ranges from 0.5 to 1.5 m deep. Extraction ceased in 2020, and a depth of around 2–3 m of peat was harvested in total, leaving a residual peat layer of 1 m on average; however, the depth ranges from 0.4 to 2 m. Below the peat, there is a clay mineral soil. Afforestation of the site began in 2022. Prior to afforestation, the site was devoid of vegetation except around ditches. The site was fertilised with 7 t ha^−1^ of wood ash in January 2022. The main nutrients in the wood ash by dry matter composition were calcium (22%), potassium (2.8%), phosphorus (0.73%), and boron (0.02%). Ditches were cleaned in May 2022 to improve drainage. Two‐year‐old Scots pine ( *Pinus sylvestris* ) saplings were planted in June 2022 with a planting density of approximately 1800–1900 trees per hectare. Natural emergence of pines will likely raise the density to around 2050 trees per hectare.

Peat samples were taken at the four chamber locations prior to afforestation (Figure 1) at multiple depths (Table 1). The dry bulk density of the samples ranged from 0.12 to 0.24 g cm^−3^, with an overall sample mean of 0.16 g cm^−3^ (Table 1). The mean total organic carbon (TOC) content, determined according to standard EN 15936:2022 (dry combustion), of the upper 0–20 cm layer was 55% ± 1.6%. TOC values across all layers were typically between 52% and 56%, with the two lower values of 38% and 36% found close to the deepest depth of peat at points E (45 cm) and W (95 cm), respectively. The mean total N content, determined according to standard EN 13342:2000 and EN 13654:2002, between 0 and 20 cm was 19.5 ± 7.1 g kg^−1^ and values ranged between 16 and 26 g kg^−1^ across all layers sampled. The mean C/N ratio of the 0–20 cm layer was 31.8 ± 11.7, which was higher than the mean values of between 40–60 cm and 80–100 cm of 21.5 ± 5.1 and 24.8 ± 5.7, respectively. The groundwater was typically acidic, where the means of the groundwater pH measured at the chamber plots ranged between 5.1 and 5.5.

**TABLE 1:** Summary of the peat geochemical properties at the study site Naarasneva. Sampling sites N, E, S, and W correspond to the chamber sampling positions in Figure 1.

Site	Peat depth [cm]	Sample depth [cm]	Dry BD [g cm^−3^]	TOC [%]	N [g kg^−1^]	C/N ratio
N	100	0–20	0.16	55	17	39
		50	0.13	56	23	25
		100	0.16	52	24	22
E	45	0–20	0.20	54	26	22
		45	0.22	38	25	15
S	> 100	0–20	0.17	55	17	33
		50	0.13	55	25	22
		85–100	0.16	36	16	22
W	95	0–20	0.18	56	18	34
		40–50	0.12	56	25	23
		80–95	0.24	52	18	30

Abbreviations: BD, bulk density; C/N ratio, carbon to nitrogen ratio; N, nitrogen; TOC, total organic carbon.

### 2.2 Satellite Data

Sentinel‐2 satellite observations were used to investigate the revegetation of the site during afforestation. The leaf area index (LAI) and normalised difference vegetation index (NDVI) were estimated using the satellite tools Python package (Nevalainen 2022). The satellite tools package retrieves bottom‐of‐atmosphere level‐2A Sentinel products via Google Earth Engine (Gorelick et al. 2017), filters the observations using the band scene classification data, and calculates LAI using the algorithm developed for the Sentinel Application Platform (Weiss et al. 2016). Satellite data were retrieved for the months April to October for the years 2021–2024. The area used for assessing LAI and NDVI corresponds to the area within the 90% contour of the EC flux footprint climatology (Figure 1). In situ observations of LAI were performed in 2024 using a LI‐COR LAI‐2200C (LI‐COR Biosciences, Lincoln, NE, USA), and we compare them to the satellite‐derived estimates.

### 2.3 Eddy Covariance and Meteorological Measurements

EC fluxes were measured at Naarasneva between August 2021 and December 2024 (Figure 1). The EC system measured at 3 m above the ground and consisted of a Metek uSonic‐3 Scientific sonic anemometer (Metek GmbH, Elmshorn, Germany) and a LI‐COR LI‐7200RS closed‐path CO_2_/H_2_O gas analyser. EC fluxes of sensible heat (H), latent heat (LE), and CO_2_ were processed using EddyUH (Mammarella et al. 2016) using standard flux processing techniques and corrections (Sabbatini et al. 2018). The processing and quality filtering are described in detail in Appendix A.

Meteorological measurements were collected on a mast adjacent to the EC system. The variables measured include air temperature (TA) and relative humidity (RH) (Vaisala HMP110; Vaisala Oyj, Vantaa, Finland), incoming and outgoing shortwave (SW_in_, SW_out_) and longwave (LW_in_, LW_out_) radiation (Kipp and Zonen CNR 4 net radiometer, OTT Hydromet B.V., Delft, Netherlands), and photosynthetically active radiation (PAR) (Kipp and Zonen PQS 1). Additional measurements collected at the site include soil temperature (TS) at 5 cm (PT100, Nokeval Oy, Nokia, Finland) and soil moisture (SWC) at 10 cm (Delta‐T ML3 ThetaProbe, Delta‐T, Cambridge, UK), and ground heat flux (G) (Hukseflux HFP01SC, Hukseflux Thermal Sensors B.V., Delft, Netherlands). Groundwater levels (WL) were measured at the four chamber measuring locations (Figure 1) with Odyssey capacitance water level loggers (Dataflow Systems Ltd., Christchurch, New Zealand), but data were only usable from plots N and S. Data from FMI weather stations were used as ancillary data and for gapfilling missing data in weather variables. Observations of TA, RH, pressure (PA), precipitation (P), and snow depth were taken from Alajärvi Möksy (FMI ID: 101533, 27 km away), and incoming shortwave radiation from Seinäjoki Pelmaa (FMI ID: 101486, 81 km) was used. The FMI data were aggregated from a 10‐ to 30‐min frequency to match the averaging period of the EC data.

Missing records of the net ecosystem exchange (NEE) of CO_2_ were gap‐filled with machine learning using gradient boosted regression (XGBoost) (Chen and Guestrin 2016). We decided to use XGBoost rather than the commonly used marginal distribution sampling (MDS) approach because MDS systemically overestimates emissions and underestimates sequestration of CO_2_ in northern (latitude > 60°) sites (Vekuri et al. 2023). The gap‐filling method is described in detail in Appendix A. Annual fluxes were calculated using the cumulative sum of the observed and gap‐filled data. NEE was partitioned into ecosystem respiration (R_eco_) and gross primary production (GPP) using the nighttime approach of Reichstein et al. (2005).

### 2.4 Energy Fluxes

We investigated changes in surface albedo and the Bowen ratio with changes in land cover during the study period. Albedo was calculated as the ratio of SW_out_/SW_in_ as measured by the net radiometer at the study site using midday hours between 10:00 and 16:00. The Bowen ratio (*β*) was calculated as the ratio of H/LE, where H and LE were measured by the eddy covariance system.

We also estimated the radiative forcing from changes in surface albedo. We used the parameterisation given by Bright and O'Halloran (2019):

(1) $$RF_{\Delta \alpha }\left(t\right)=\frac{1}{12}\sum_{m=1}^{m=12}-SW_{\text{in}\left(m,t\right)}\sqrt{T_{a\left(m,t\right)}}\Delta \alpha_{\left(m,t\right)}$$

where $RF_{\Delta \alpha }$ is the annual radiative forcing due to a change in surface albedo at year *t* and the units are W m^−2^, SW_in_ is incoming shortwave radiation measured at the surface at year *t* and month *m*, *T*_*a*_ is the upward atmospheric transmittance and Δα is the change in surface albedo. Assuming downward and upward transmittances to be equal, *T*_*a*_ was approximated as the ratio of SW_in_/SW_TOA_ where SW_TOA_ is incoming shortwave radiation at the top of atmosphere (TOA). SW_in_ was derived from monthly mean midday measurements at the site surface and monthly mean SW_TOA_ was calculated from daily approximations of SW_TOA_ after Duffie and Beckman (2013) (described in Supporting Information S1). A reference albedo needs to be defined to calculate Δ*α*, which in our case represents the albedo if no land cover change occurred (i.e., bare peat). Since we did not have a full year of measurements prior to afforestation, the reference albedo was defined using measurements from 2021 and 2022 before any clear land cover changes were evident. Furthermore, since $RF_{\Delta \alpha }$ is sensitive to the length of the snow cover period, particularly in the spring season, we removed months affected by snow cover (i.e., set Δ*α* = 0) to focus on the effect of changing land cover in snow‐free months (May to September). This decision is discussed later in the Discussion and in Supporting Information S1.

To compare the impact of albedo change with GHGs, we also present a CO_2_‐equivalent metric for albedo change similar to previous studies:

(2) $$\begin{array}{c} \text{EESF}/\mathrm{TH}=\frac{RF_{\Delta \alpha }}{k_{CO_{2}}A_{E}\mathrm{AF}}\frac{1}{\mathrm{TH}} \end{array}$$

where EESF/TH is the CO_2_ emissions (or removal) equivalent of shortwave forcing (EESF) due to a change in albedo divided by a time horizon (TH) to give emissions in kg CO_2_‐eq m^−2^ year^−1^, $k_{CO_{2}}$ is the global mean radiative efficiency of CO_2_ ($1.76\times 10^{-15}$ W m^−2^ kg^−1^), $A_{E}$ is the surface area of earth ($5.101\times 10^{14}$ m^2^), AF is the average airborne fraction of CO_2_ and represents the proportion of anthropogenic emissions of CO_2_ that remains in the atmosphere after a set time period, and TH is the time horizon. We used a TH of 100‐years so that EESF can be compared easily with the other GHGs in the study (see Section 2.7); however, we acknowledge there are issues with this TH, as the land cover is transient because of the gradual forest transition. This is complicated by the fact that the energy change is instantaneous and does not have a tail like the radiative forcing from CO_2_ emissions (Betts 2000; Bright et al. 2015; Bright and Lund 2021; Carrer et al. 2018; Muñoz et al. 2010). In addition, a longer time horizon, as used here, underestimates the impact of an albedo change in the short‐term (Bright and Lund 2021; Carrer et al. 2018). Since a 100‐year TH was chosen, the AF parameter was set to 0.48, which is the remaining fraction given by the Bern carbon cycle model (Joos et al. 2001) when integrating over that time period (Carrer et al. 2018). The annual uncertainty of ESSF/TH was calculated as the sum of aggregated uncertainty from the variance of observed albedos for a month and from the variance of the reference albedo, both of which were propagated through Equations (1) and (2).

### 2.5 Chamber Measurements

Fluxes of methane (CH_4_) and nitrous oxide (N_2_O) were measured using the manual chamber method at four plots approximately 50 m from the EC tower in cardinal directions: north (N), east (E), south (S), and west (W) (Figure 1). At the four plots, fluxes were measured from the ditch and at four strip subplots spaced at intervals of approximately 2.5 m from the ditch to the middle of the strip (~10 m). Some variation in measuring point spacing occurred because of terrain variation. A closed and opaque aluminium cylindrical chamber with a volume of 0.02217 m^3^ was used and which included a fan to mix the air inside the chamber headspace. The chamber was placed directly onto the peat soil for strip measurements, and the same chamber was floated using a polystyrene mould for ditch measurements. The chamber was placed directly onto the ditch if there was not enough water present to float the chamber. Mixing ratios were measured at a 1 s frequency using portable gas analyser models LI‐COR LI‐7810 for CH_4_ and LI‐COR LI‐7820 for N_2_O, except for one sample trip in August 2023 where an ABB LGR‐ICOS M‐GGA‐910 (ABB Inc., Quebec, Canada) was used to measure CH_4_. Chamber closure times varied between 120 and 180 s, where each closure was manually screened for disturbances at the beginning or end of the measurement and was excluded accordingly. Measurements of CH_4_ were conducted between July 2021 and October 2024 and measurements of N_2_O were conducted between July 2022 and October 2024. A total of 38 measurement campaigns were performed for CH_4_ with a median time between measurements of 15 days (min 8, max 313). Fewer measurements were performed for N_2_O, with a total of 18 measurement campaigns and a median time between measurements of 32 days (min 13, max 213). The long sampling gap for CH_4_ and N_2_O was between late 2023 and early 2024 and was because of a funding gap between projects. During the winter measurements (25.01.2023, 22.02.2023, and 23.03.2023), the two strip subplots closest to the middle of the strip were excluded from all four measurement plots (N, E, S, and W), resulting in six fewer measurements. Fewer points were sampled because of the short winter days and freezing temperatures which caused the gas analyser batteries to rapidly deplete and did not allow more measurements per day. This exclusion applies to both CH_4_ and N_2_O gases. Fluxes of CH_4_ (FCH_4_) and N_2_O (FN_2_O) were calculated from the change in gas concentration over time using the slope fitted by linear regression. Exponential fits were also tested but which resulted in many unrealistic fluxes for many measurements. This was particularly the case for measurements with small changes in concentration, as found previously (e.g., Korkiakoski et al. 2017). Positive flux values signify emission from the ecosystem to the atmosphere.

Fluxes were aggregated annually by first taking monthly means of strip and ditch fluxes. Gaps of 1 month were linearly interpolated first, followed by filling the year‐season mean, overall month mean, and overall season mean if gaps remained. Longer linear interpolation was not used because of the incomplete and unequal temporal sampling. Annual means of strip and ditch fluxes were taken from the gap‐filled timeseries. Finally, the weighted mean of annual strip and ditch fluxes was taken using values of 95.1% and 4.9%, respectively, derived from the areal coverage of those land features from the mean EC flux footprint climatology for the reported estimate of the annual flux of CH_4_ and N_2_O. The uncertainty of the annual totals was calculated using the aggregated variance from measured data and gap‐filled data. The uncertainty of measured data was the variance of the samples used to calculate the monthly mean, and the uncertainty of the gap‐filled data was the variance of the measurements used to calculate the gap‐filled mean. When taking the uncertainty of the annual mean only the number of measured values was used when averaging, since artificial replicates should not contribute to a reduction of uncertainty, that is

(3) $$\begin{array}{c} {\mathrm{SE}}_{\text{Annual}}=\frac{\sqrt{\sum \sigma_{\text{Measured}}^{2}+\sum \sigma_{\text{Gapfilled}}^{2}}}{n_{\text{Measured}}} \end{array}$$

where $\sigma_{\text{Measured}}^{2}$ is the variance of measured values, $\sigma_{\text{Gapfilled}}^{2}$ is the variance of gap‐filled values, $n_{\text{Measured}}$ is the number of measured values, and SE_Annual_ is the standard error of the mean annual chamber flux.

### 2.6 Water Sampling and Lateral C Fluxes

Water samples were taken at the four chamber plots between May 2021 and October 2024 (Figure 1). The water samples were extracted from 0.5 and 1 m slotted dipwells by purging the well and letting it refill. If the well did not refill in a reasonable time, the first pumped water was sampled. Additional water samples were collected from the adjacent ditch at the chamber sampling points. In total, *n =* 54 samples of soil water and *n* = 18 ditch water samples were analysed for dissolved organic carbon (DOC) concentrations according to standard SFS‐EN 1484:1997. Measured regional‐specific discharge (L^−1^ s^−1^ km^2^) from small catchments (Kainastonluoma, Kaidesluoma, and Pahkaoja) was collected from the Hertta Database (2025) and was used to estimate discharge values from the Naarasneva site. The Hertta database is managed by the Finnish Environment Institute (SYKE). DOC lost via lateral water flows (FDOC) was estimated using measured DOC concentrations from porewater and ditch water and estimates of daily specific discharge. Water sampling was done approximately at monthly intervals, and the sampled concentration was assumed to represent lateral concentration values until the next sampling round. For FDOC, we mainly used samples taken from the ditch network as they represent lateral C leaving from the afforested site. If ditch water was lacking from the sampling round, porewater samples were used to estimate ditch water concentration using a linear relationship ($\mathrm{DO}C_{\text{ditch}}=0.1773\times \mathrm{DO}C_{\text{porewater}}+38.472;R^{2}=0.72$). The relationship was constructed from periods when samples of ditch and porewater were both present, and we also included samples from control plots at the site that are otherwise not included in the analysis to increase the predictive power. Annual FDOC totals were estimated by taking the cumulative sum of estimated daily FDOC. The annual uncertainty was aggregated from the uncertainty of daily FDOC, which was calculated from the variance of DOC measurements and the variance of the specific discharge from the three catchments.

### 2.7 Annual Net Ecosystem Carbon Balance and Radiative Balance

We present estimates of the net ecosystem carbon balance (NECB), which represents the full C balance of the site. For our site, the NECB is defined as:

(4) $$\begin{array}{c} \text{NECB}=-\mathrm{NEE}-{\mathrm{FCH}}_{4}-\text{FDOC} \end{array}$$

where NEE is the annual estimate of CO_2_ flux derived from EC measurements, FCH_4_ is the annual CH_4_ flux derived from chamber observations, and FDOC is the annual lateral aqueous loss of C. The NECB uses the notation that a positive value is an ecosystem sink of C and a negative value is an ecosystem source (Chapin et al. 2006). All terms are expressed in units of g C m^−2^ year^−1^. In our case, there were no external additions of C that needed to be accounted for (e.g., application of C containing fertiliser).

The radiative balance attempts to weigh the emissions of gases and ecosystem albedo on the basis of their effect on the Earth's energy budget over a defined time horizon (Neubauer 2021). Here, we calculate the radiative balance as:

(5) $$\begin{array}{c} \text{Radiative balance}=\mathrm{NEE}+{\mathrm{CH}}_{4}{\mathrm{GWP}}_{100}.{\mathrm{FCH}}_{4}\\ \;+N_{2}{\text{OGWP}}_{100}.{\mathrm{FN}}_{2}O+\text{EESF}/\mathrm{TH} \end{array}$$

where NEE and FCH_4_ have the same definitions as in Equation (4) except they are in units of g CO_2_ m^−2^ year^−1^ and g CH_4_ m^−2^ year^−1^, respectively, FN_2_O is the annual flux of N_2_O derived from chamber observations in g N_2_O m^−2^ year^−1^, and EESF/TH is the CO_2_‐eq effect of albedo change in g CO_2_‐eq m^−2^ year^−1^. The emissions of FCH_4_ and FN_2_O are converted in CO_2_‐equivalents (CO_2_‐eq) by multiplying the annual totals by their 100‐year global warming potentials (GWP_100_) of CH_4_GWP_100_ = 27 and N_2_OGWP_100_ = 273, respectively, and therefore the balance is in units of g CO_2_‐eq m^−2^ year^−1^ (IPCC 2021). GWPs of 100 years were chosen since they form a compromise between long and short‐lived emissions of GHGs. For changes in the radiative balance, that is, the radiative forcing (Neubauer 2021), a negative sign indicates a cooling effect and a positive sign indicates a warming effect. We also present the CO_2_‐eq GHG balance, which is the radiative balance without EESF/TH. The annual uncertainties of the NECB, radiative balance, and CO_2_‐eq GHG balance were calculated as the square root of the sum of squared error of the variables in the equations.

## 3 Results

### 3.1 Satellite Data and Afforestation

**FIGURE 2:**
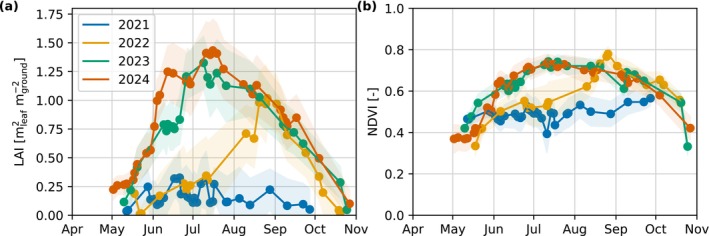
Sentintel‐2 satellite‐derived estimates of (a) leaf area index (LAI) and (b) normalised difference vegetation index (NDVI) over the growing season. The points are the mean and the shading is one standard deviation of pixels within the 90% eddy covariance flux footprint climatology (Figure 1) for each satellite capture.

The median revisit time of usable satellite images (i.e., after quality filtering) was 4.0 days, and the longest within‐season gap was 29.0 days. The number (*n*) of usable captures per year was 20, 23, 30, and 31 for the years 2021–2024, respectively. The satellite‐derived LAI compares well to field observations of LAI collected in 2024 (Figure S2.1); however, the field observed data have a wider spread and a slightly different seasonality. Satellite LAI and NDVI values increased year‐on‐year from 2021 to 2023 and then remained steady in 2024, where, after 2021, there is also a clear seasonal signal in both metrics corresponding to the growing season (Figure 2). The LAI during the summer peak increased from around 0.2 $m_{\text{leaf}}^{2}\;m_{\text{ground}}^{-2}$ in 2021 to 1.2 $m_{\text{leaf}}^{2}\;m_{\text{ground}}^{-2}$ in 2024, and similarly for NDVI from around 0.50 to 0.70 from 2021 to 2024, respectively. The effect of fertilisation in 2022 was also captured with a sharp increase in both LAI and NDVI towards the end of August in 2022 compared to the year prior. Increases in LAI and NDVI were mostly due to the emergence of several plant species after fertilisation at the site. The most abundant species at the site in 2024 were cottongrass ( *Eriophorum vaginatum* ), reedgrass (*Calamagrostis phragmitiodes*), willowherb ( *Chamaenerion angustifolium* ), and downy birch ( *Betula pubescens* ).

### 3.2 Study Period Weather

**FIGURE 3:**
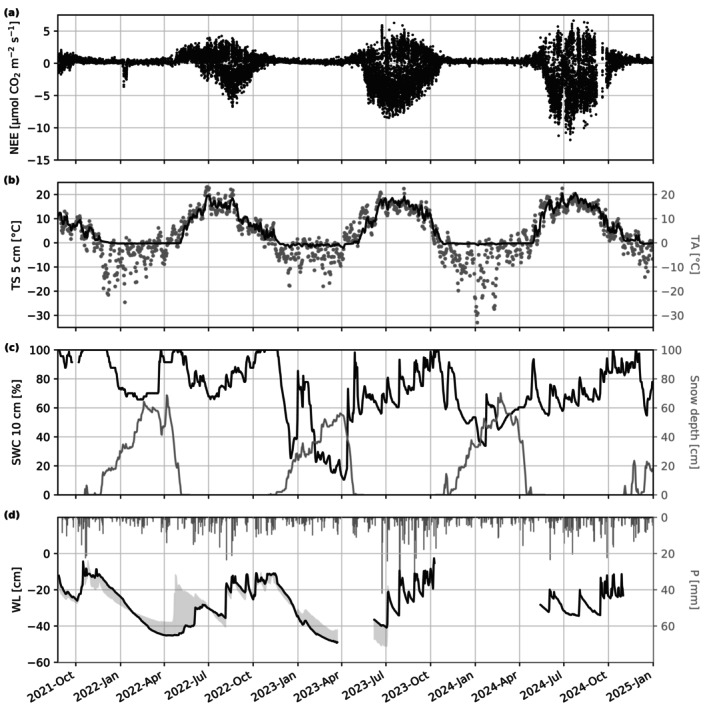
Timeseries of key variables over the study period: (a) half‐hourly net ecosystem exchange (NEE) of CO_2_, (b) daily mean soil temperature (TS) at 5 cm and air temperature, (c) daily mean soil water content at 10 cm (SWC) and snow depth, and (d) daily mean water level (WL) and daily precipitation (P) totals. In plot (d) the solid line is the WL in plot N which was selected as it had the most complete temporal coverage; the shading indicates the range of data collected from plot N and S.

The length of the seasons varied year‐to‐year (Table 2). The seasons were defined according to meteorological conditions following the FMI definition. Spring begins when the daily mean TA remains above 0°C for 10 days consecutively, summer begins when the daily mean TA exceeds 10°C for five consecutive days and thereafter does not fall below 10°C for five consecutive days, autumn begins when the daily mean TA falls below 10°C and thereafter does not exceed 10°C for more than 2 days, and winter begins when the daily mean TA falls below 0°C and remains below 0°C for five consecutive days. The lengths of spring and autumn were typically short compared to winter and summer, and they could be remarkably short, where autumn 2023 and spring 2024 were 16 and 17 days, respectively. The lengths of winter were similar in 2021 and 2022, whereas the winter of 2023–2024 was longer by around 48 days. Year 2024 had a longer summer and a higher mean seasonal temperature compared to the two previous years. This year also had earlier snowmelt and higher temperatures in the spring and early summer, as well as an absence of precipitation (Figure 3).

**TABLE 2:** The start date of each season (day.month), the number of days (*n*) in parentheses, and the mean air temperature (TA) for each season.

Year	Spring		Summer		Autumn		Winter		Annual
	Start (*n*)	TA [°C]	Start (*n*)	TA [°C]	Start (*n*)	TA [°C]	Start (*n*)	TA [°C]	TA [°C]
1990–2024	06.04 (56)	6.0 ± 1.9	02.06 (109)	14.1 ± 1.0	20.09 (41)	4.7 ± 1.1	01.11 (155)	−4.8 ± 1.9	3.6 ± 0.8
2021					31.08 (81)	5.2	21.11 (140)	−5.6	
2022	11.04 (40)	4.5	22.05 (116)	14.1	16.09 (59)	5.1	15.11 (141)	−4.9	4.0
2023	06.04 (65)	6.5	11.06 (111)	14.6	01.10 (16)	3.7	18.10 (189)	−6.2	3.3
2024	25.04 (17)	4.2	13.05 (130)	14.9	21.09 (39)	5.3	31.10 (—)		3.8

*Note:* Season length was determined following the Finnish Meteorological Institute (FMI) definition where autumn is when the daily mean TA stayed below 10°C for autumn and 0°C for winter, and when the temperature stayed above 0°C and 10°C for spring and summer, respectively. The long‐term 1990–2024 means were derived from FMI gridded data.

### 3.3 CO_2_ Fluxes

NEE was highly seasonal and followed the typical northern European pattern (Figure 3). Generally, only net emission of CO_2_ was measured in winter and at the beginning of spring, with the transition to net uptake occurring from mid‐spring to early summer each year. An anomaly was measured in January 2022 where there was a net uptake of CO_2_ following the application of wood ash fertiliser. Wood ash typically contains high amounts of calcium oxide which can undergo carbonation using atmospheric CO_2_ to form calcium carbonate (e.g., Wu et al. (2024)). The strength of uptake and emission of NEE increased year‐on‐year over the study period (Figure 3). Medians of daily median observed daytime and (nighttime) NEE during summer were −0.3 (2.0) μmol CO_2_ m^−2^ s^−1^ in 2022, −3.1 (2.5) μmol CO_2_ m^−2^ s^−1^ in 2023, and −3.5 (3.1) μmol CO_2_ m^−2^ s^−1^ in 2024.

**FIGURE 4:**
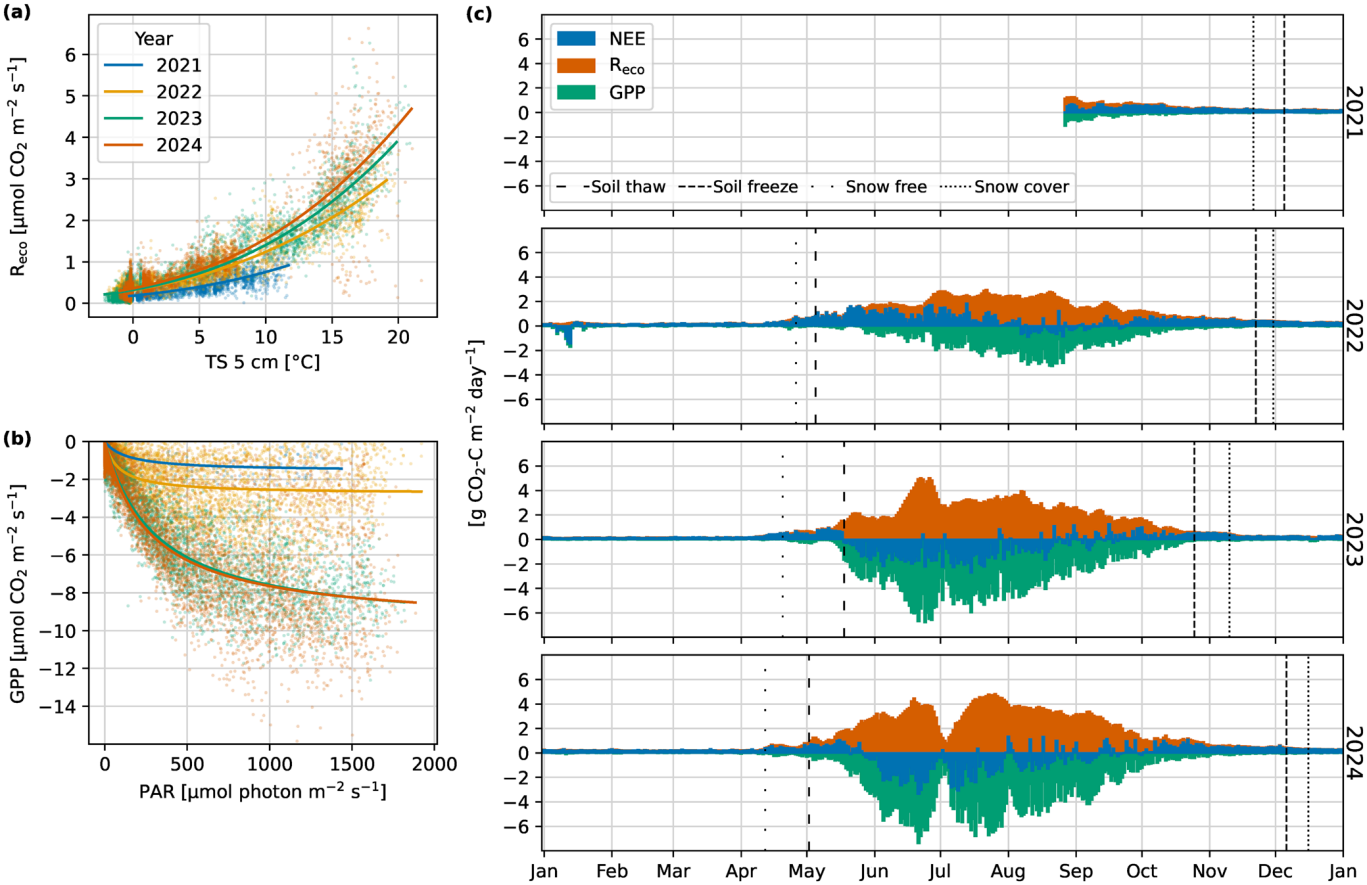
(a) Response of ecosystem respiration (R_eco_) to soil temperature (TS) at 5 cm annually and (b) light response of gross primary production (GPP) to photosynthetically active radiation (PAR) during the summer season. Data points in (a) and (b) are half‐hourly averages and the lines are mean annual fits for trend visualiation. (c) Daily totals of gap‐filled net ecosystem exchange (NEE) of CO_2_, R_eco_, and GPP for each study year.

The response of R_eco_ to TS increased for each year from 2021 to 2024, and the summer GPP uptake in response to light exposure similarly increased (Figure 4a,b). The daily balances of partitioned CO_2_ fluxes show that the typical periods with photosynthesis were between mid‐May and the end of October (Figure 4c). GPP progressively increased during the summer of 2022 where the monthly mean of daily GPP increased from −0.82 g CO_2_‐C m^−2^ day^−1^ in June 2022 to −2.50 g CO_2_‐C m^−2^ day^−1^ in August 2022. The site only had a few days with net uptake of CO_2_ in August 2022 and was otherwise a net source. Higher rates of GPP started earlier in 2023 compared to 2022, where the monthly mean GPP was −1.31 g CO_2_‐C m^−2^ day^−1^ in May 2023 and the maximum uptake was reached in June 2023 at −4.59 g CO_2_‐C m^−2^ day^−1^. The site was also a net sink of CO_2_ earlier in 2023 than in 2022, where net uptake was observed from mid‐May to late August 2023. Mean monthly GPP was slightly lower in May 2024 at −1.33 g CO_2_‐C m^−2^ day^−1^ and reached a higher maximum uptake in June at −5.08 g CO_2_‐C m^−2^ day^−1^. The site was a net sink between June and the start of September in 2024. Occasional occurrences of positive NEE during the middle of summer in 2023 and 2024 were mostly caused by reduced incoming radiation due to cloud cover. R_eco_ also increased year‐on‐year, where the mean value for each summer period was 2.11, 2.90, and 3.17 g CO_2_‐C m^−2^ day^−1^ in 2022, 2023, and 2024, respectively. Figure 4c shows low NEE uptake and reduced rates of R_eco_ and GPP around the beginning of July 2024. Values began to trend lower after a sustained period of above‐average temperatures and low rainfall in May and June (Figure 3). However, the highest values of NEE (i.e., reduced uptake) during this period were measured on days with precipitation and lower temperatures.

**FIGURE 5:**
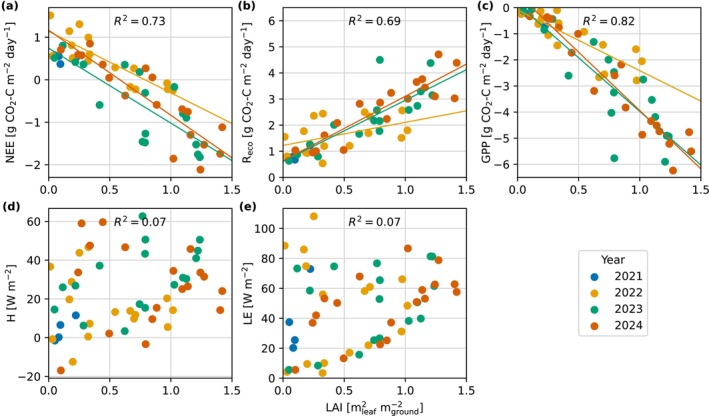
Mean weekly leaf area index (LAI) plotted against mean weekly (a) net ecosystem exchange (NEE), (b) ecosystem respiration (R_eco_), (c) gross primary production (GPP), (d) sensible heat flux (H), and (e) latent heat flux (LE). In each subplot the coefficient of determination (*R* ^2^) between LAI and the respective variable is presented, and in plots (a) to (c) the lines are the linear trends for each year.

Changes in the CO_2_ fluxes had linear relationships with changes in LAI (Figure 5a–c). Weekly mean LAI and NEE had a coefficient of determination (*R* ^2^) of 0.73, and the relationship between LAI and the partitioned fluxes improved for GPP (*R* ^2^ = 0.82) but not R_eco_ (*R* ^2^ = 0.69). Linear regression slopes of all data indicate that per 1 $m_{\text{leaf}}^{2}m_{\text{ground}}^{\text{−}2}$ increase in LAI there is a −4.04 g CO_2_‐C m^−2^ day^−1^ change in GPP and a 2.19 g CO_2_‐C m^−2^ day^−1^ change in R_eco_. The steepness of LAI to GPP slope also increased over time, with slopes of −2.33, −4.11, and −4.47 $g\;{\mathrm{CO}}_{2}\text{‐}C\;m_{\mathrm{leaf}}^{-2}\;{\mathrm{day}}^{-1}$ fit for 2022, 2023, and 2024, respectively (Figure 5a–c). Similarly, the slopes of LAI to R_eco_ became steeper over time with values of 0.88, 2.34, and 2.46 $g\;{\mathrm{CO}}_{2}\text{‐}C\;m_{\mathrm{leaf}}^{-2}\;{\mathrm{day}}^{-1}$ for 2022, 2023, and 2024, respectively.

### 3.4 Energy Fluxes

**FIGURE 6:**
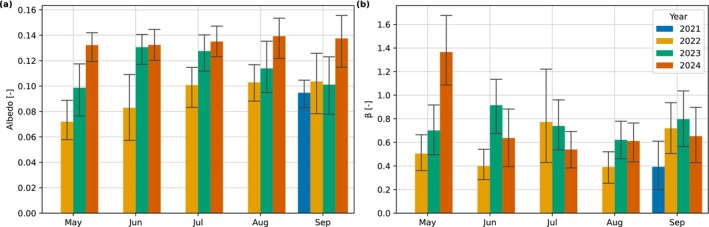
(a) Monthly median albedo calculated from observed incoming and outgoing shortwave radiation and (b) monthly median mid‐day Bowen ratios (*β*) calculated from observed sensible and latent heat flux data. Timesteps between 10:00 and 16:00 were used to calculate the medians and the uncertainty interval is the interquartile range. Monthly albedos of all months are presented in Figure S2.3.

The monthly median midday albedo measured at the site with the net radiometer ranged from 0.07 to 0.14 (Figure 6a). The median values of albedo when the site had bare peat in May and June 2022 were 0.07 and 0.08, respectively. From then, there is a progressive yearly increase in albedo for each month and the summertime values in 2024 were between 0.13 and 0.14. There were no clear patterns in the Bowen ratio (*β*), the ratio of H to LE, that can be simply related to afforestation (Figure 6b). Rather, the partitioning between H and LE appears to be driven by water availability as indicated by the weak Pearson's correlation between monthly *β* and WL (*r* = 0.25). For example, the highest *β* was observed in May 2024, a year when there was earlier loss of the winter snowpack (Figure 3), low monthly precipitation (8.5 mm, long term mean: 44.8 mm), and higher than average temperatures (monthly mean TA: 11.2°C, long‐term monthly mean TA: 8.7°C). Monthly diurnal plots of H, LE, and *β* are presented in Figure S2.2. There are no clear interannual patterns with the relationships of LAI and H and LE, but there is clear hysteresis which relates to seasonality (Figure 5d,e).

The annual $RF_{\Delta \alpha }$ neglecting snow effects decreased yearly at the site and corresponded to yearly increases in surface albedo (Table S1.3). $RF_{\Delta \alpha }$ was calculated to be −0.2 ± 1.0, −1.6 ± 1.0, and −2.9 ± 1.1 W m^−2^ for 2022, 2023, and 2024, respectively (CO_2_‐eq values presented in Table 3 and Section 3.7). Monthly albedo statistics and reference albedos used to calculate $RF_{\Delta \alpha }$ are presented in Tables S1.1 and S1.2, respectively.

### 3.5 CH_4_ and N_2_O Fluxes

**FIGURE 7:**
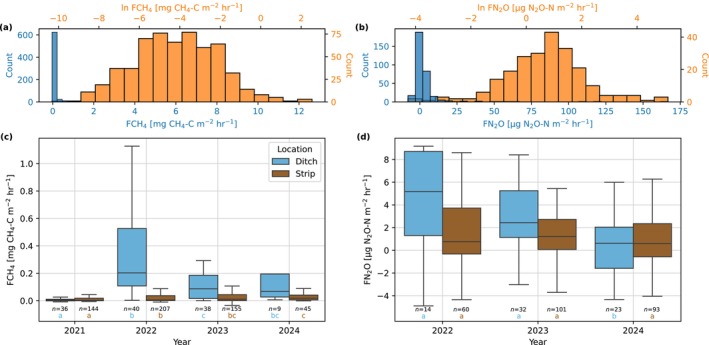
Observed and natural logarithm transformed distributions of (a) methane fluxes (FCH_4_), and (b) nitrous oxide fluxes (FN_2_O) measured using manual chambers. Annual boxplots of (c) FCH_4_ and (d) FN_2_O, split by the sampling location of ditch or strip. The number of values (*n*) used is printed below each box as well as letter codes that indicate significant differences (*p* < 0.05) according to the Wilcoxon rank‐sum test. Values beyond the boxplot whiskers in (c) and (d) have been omitted to enhance the view of most of the distribution (full figure presented in Figure S2.4). A timeseries of observations of FCH_4_ and FN_2_O is presented in Figure S2.5.

The distribution of FCH_4_ was right‐skewed, where the overall mean was 0.122 mg CH_4_‐C m^−2^ h^−1^ and overall median 0.009 mg CH_4_‐C m^−2^ h^−1^ (Figure 7a). The median emission of ditches (0.069 mg CH_4_‐C m^−2^ h^−1^) was nearly an order of magnitude greater than the median of strip emissions (0.007 mg CH_4_‐C m^−2^ h^−1^). Measured strip FCH_4_ was mostly positive, where net emission occurred in 85% of observations. There was variability in FCH_4_ annually (Figure 7c); however, interannual comparisons are complicated by unequal temporal sampling. Ditch emissions were low in 2021 (half‐year of measurements), increased in 2022 and then declined to 2024, where the corresponding median annual emission rates were 0.005, 0.203, 0.087, and 0.069 mg CH_4_‐C m^−2^ h^−1^. Median strip emission rates increased in each subsequent measurement year from 2021 to 2024 with median emission rates of 0.004, 0.006, 0.011, and 0.018 mg CH_4_‐C m^−2^ h^−1^, respectively. To check for significant differences, we applied the Wilcoxon rank‐sum test since the data have a non‐normal distribution and the presence of negative flux values limits a test on log‐transformed data. For ditch FCH_4_, the year 2021 was significantly different (*p <* 0.05) from all other years and 2022 was significantly different to 2023. For strip FCH_4_, 2021 was also significantly different from all other years and 2022 was significantly different to 2024.

FN_2_O also had a right‐skewed distribution where the overall mean and median were 4.0 and 1.1 μg N_2_O‐N m^−2^ h^−1^, respectively (Figure 7b). The median of ditch FN_2_O (1.8 μg N_2_O‐N m^−2^ h^−1^) was greater than strip FN_2_O (0.9 μg N_2_O‐N m^−2^ h^−1^). Uptake of N_2_O was measured in 30% of strip measurements and in 21% of ditch measurements. Median annual strip FN_2_O ranged between 0.6 and 1.2 μg N_2_O‐N m^−2^ h^−1^ and did not show an increasing or decreasing trend, whereas median ditch fluxes progressively decreased year‐on‐year with 2022, 2023, and 2024 at 5.2, 2.4, and 0.6 μg N_2_O‐N m^−2^ h^−1^, respectively (Figure 7d). Applying the same approach as for FCH_4_, the results of the Wilcoxon rank‐sum test showed that no years were significantly different for strip FN_2_O, whereas for ditch FN_2_O the year 2024 was significantly different from 2022 and 2023.

Relationships between meteorological and soil variable drivers of FCH_4_ and FN_2_O, which overall were weak, are presented in Supporting Information S3.

### 3.6 DOC Concentrations and Lateral Fluxes

**FIGURE 8:**
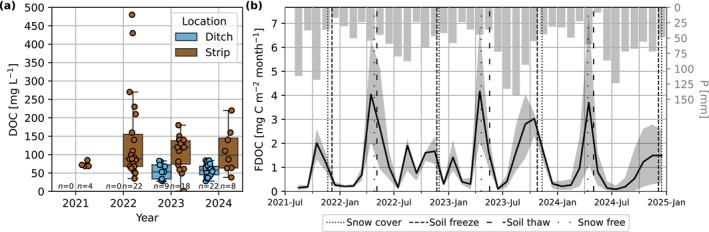
(a) Boxplot of dissolved organic carbon (DOC) concentrations and (b) timeseries monthly estimates of DOC leaching (FDOC) and precipitation (P, right axis). The shaded area around FDOC is the minimum and maximum prediction interval. A timeseries of DOC concentrations is presented in Figure S2.6.

Strip concentrations of DOC ranged from 1 to 480 mg L^−1^ and had an overall mean and median of 116 and 89 mg L^−1^, respectively (Figure 8a). The annual medians of strip DOC concentrations increased from 70 to 88 to 120 mg L^−1^ from 2021, 2022, and 2023, respectively, and then declined in 2024 to 98 mg L^−1^ (note that only a few samples were collected in 2024 for the strip [*n* = 8]; Figure 8a), but no years were significantly different from each other according to the Wilcoxon rank‐sum test. Ditch DOC concentrations ranged from 28 to 84 mg L^−1^ and had an overall mean of 52 mg L^−1^ and a median of 53 mg L^−1^. Samples of ditch water were only collected in 2023 and 2024 (Figure 8a) and these years were also not significantly different. Losses of DOC were driven by discharge derived from snowmelt or sustained high precipitation (Figure 8b). The low variation of DOC in ditch water also indicates that the DOC losses are derived from mobilised strip C.

### 3.7 Annual Totals, Carbon Balance and Radiative Balance

The performance of XGBoost gapfilling NEE was good; the test set performance scores of the model ensemble mean were *R* ^2^ = 0.94, RMSE = 0.4494 μmol CO_2_ m^−2^ s^−1^, and mean bias = −0.0038 μmol CO_2_ m^−2^ s^−1^. Annual totals are presented as the cumulative annual flux ± the 95% uncertainty interval. The site was a net source of CO_2_ in 2022 with an annual NEE of 144 ± 20 g CO_2_‐C m^−2^ year^−1^, followed by 2 years where it was a net sink of −36 ± 12 g CO_2_‐C m^−2^ year^−1^ in 2023 and −19 ± 19 g CO_2_‐C m^−2^ year^−1^ in 2024 (Table 3). GPP more than doubled in 2023 compared to 2022, with annual totals of −495 ± 15 and −224 ± 21 g CO_2_‐C m^−2^ year^−1^, respectively, whereas in 2024, there was a 15% increase on 2023 to −560 ± 22 g CO_2_‐C m^−2^ year^−1^. R_eco_ increased by 25% from 2022 (368 ± 8 g CO_2_‐C m^−2^ year^−1^) to 2023 (459 ± 10 g CO_2_‐C m^−2^ year^−1^) and by 18% from 2023 to 2024 (541 ± 12 g CO_2_‐C m^−2^ year^−1^).

**TABLE 3:** Annual totals of the net ecosystem exchange (NEE), ecosystem respiration (R_eco_), gross primary production (GPP), methane flux (FCH_4_), dissolved organic carbon flux (FDOC), nitrous oxide flux (FN_2_O), and the CO_2_‐equivalent (CO_2_‐eq) of shortwave forcing due to a change in surface albedo (EESF/TH).

Year	NEE	R_eco_	GPP	FCH_4_	FDOC	NECB	FN_2_O	EESF/TH^b^	CO_2_‐eq GHG balance	Radiative balance
	[g C m^−2^ year^−1^]						[mg N_2_O‐N m^−2^ year^−1^]	[g CO_2_‐eq m^−2^ year^−1^]		
2022	144 ± 20	368 ± 8	−224 ± 21	0.7 ± 0.4	15 ± 2	−160 ± 20	35 ± 30^a^	−4 ± 24	567 ± 75	563 ± 79
2023	−36 ± 12	459 ± 10	−495 ± 15	0.5 ± 0.4	18 ± 2	17 ± 12	25 ± 10	−38 ± 24	−102 ± 46	−140 ± 52
2024	−19 ± 19	541 ± 12	−560 ± 22	0.6 ± 0.6	11 ± 5	8 ± 20	18 ± 12	−67 ± 25	−42 ± 73	−109 ± 77

*Note:* The net ecosystem carbon balance (NECB) is the sum of the carbon (C) flux terms NEE, CH_4_, and FDOC (Equation 4), CO_2_‐eq greenhouse gas (GHG) balance is the sum of the gaseous fluxes NEE, FCH_4_ and FN_2_O, and radiative balance is the CO_2_‐eq GHG balance including EESF/TH (Equation 5). For all terms except NECB, a positive sign indicates an emission from the ecosystem to the atmosphere and a negative sign indicates uptake by the ecosystem. For NECB, a positive sign indicates net C uptake and negative indicates a net C loss. The 100‐year global warming potential scalars of 27 for CH_4_ and 273 for N_2_O have been used to convert emissions to CO_2_‐equivalent fluxes (IPCC 2021). The uncertainty of all totals is the 95% confidence interval.

^a^ Flux measurements of N_2_O fluxes began in July 2022 and missing values have been backfilled using mean values from the following years.

^b^ EESF/TH totals assume no change in albedo from the reference in snow affected months.

Weighted annual areal totals of CH_4_ fluxes were similar in all measurement years and ranged from 0.5 ± 0.4 to 0.7 ± 0.4 g CH_4_‐C m^−2^ year^−1^. Annual ditch fluxes were much greater than strip emissions per unit area. Mean annual emissions for ditches ranged from 4.8 ± 5.5 to 6.5 ± 7.5 g CH_4_‐C m^−2^ year^−1^ compared to 0.3 ± 0.2 to 0.4 ± 0.2 g CH_4_‐C m^−2^ year^−1^ for strip areas. Weighted annual areal totals of N_2_O fluxes ranged from 18 ± 5 to 35 ± 26 mg N_2_O‐N m^−2^ year^−1^. Ditches were also a greater source of N_2_O where mean annual emissions ranged from 138 ± 185 to 207 ± 94 mg N_2_O‐N m^−2^ year^−1^ compared to strip areas where emissions ranged from 10 ± 5 to 29 ± 26 mg N_2_O‐N m^−2^ year^−1^.

Annual totals of FDOC ranged from 11 ± 5 to 18 ± 2 g C m^−2^ year^−1^. There are no clear trends in the annual totals, though insight is limited because of the few study years.

The CO_2_‐eq total due to surface albedo changes (neglecting snow effects) was small in 2022 at −4 ± 24 g CO_2_‐eq m^−2^ year^−1^, followed by larger decreases in 2023 and 2024 of −38 ± 24 and −67 ± 25 g CO_2_‐eq m^−2^ year^−1^ (EESF/TH; Table 3).

There was a large increase in annual NECB from −160 ± 20 g C m^−2^ year^−1^ in 2022 to 17 ± 12 and 8 ± 20 g C m^−2^ year^−1^ in 2023 and 2024, respectively. The positive sign of the totals in 2023 and 2024 suggests that there may have been net ecosystem C uptake; however, the uncertainty interval indicates that there may have been small C losses as well. The CO_2_‐eq GHG balance using GWP_100_ was 567 ± 75 g CO_2_‐eq m^−2^ year^−1^ in 2022. There was a sharp reduction in 2023 to −102 ± 46 g CO_2_‐eq m^−2^ year^−1^, after which it increased slightly but remained negative to −42 ± 73 g CO_2_‐eq m^−2^ year^−1^ in 2024. The radiative balance in 2022 was 563 ± 79 g CO_2_‐eq m^−2^ year^−1^, followed by large reductions in 2023 to −140 ± 52 g CO_2_‐eq m^−2^ year^−1^ and in 2024 to −109 ± 77 g CO_2_‐eq m^−2^ year^−1^. Therefore, the radiative forcing resulting from the changes in the radiative balance fluxes due to afforestation, that is the difference between years 2023 and 2024 and 2022, was −703 and −672 g CO_2_‐eq m^−2^ year^−1^, respectively.

## 4 Discussion

### 4.1 A Quick Change From Source to Sink

Our results show that afforestation has quickly turned the study site from a source to a small sink of CO_2_. The quick change to CO_2_ sink was driven by the revegetation of the site that was accelerated by the application of wood ash fertiliser, supporting previous findings that ash‐based fertilisers cause rapid ground vegetation formation (Huotari et al. 2007, 2009). There are very few studies reporting annual NEE from afforested cutaway peatlands that can be used for comparison, and especially considering our site is in the initial stages of afforestation. Mäkiranta et al. (2007) reported soil efflux data from six cutaway peatland sites that were afforested in Finland, where the sites were abandoned 15–20 years prior to afforestation and then the forests were 18–40 years old by the time measurements began. They did not report NEE but mean annual soil CO_2_ efflux from chamber measurements ranged between 275 and 479 g CO_2_‐C m^−2^ year^−1^ and which are comparable to the range of annual values of R_eco_ we found of 368 ± 8 to 541 ± 12 g CO_2_‐C m^−2^ year^−1^. These sites had a small mean annual CH_4_ net uptake of −0.04 ± 0.02 g CH_4_ C m^−2^ year^−1^ (mean ± 1 standard deviation [SD]) and net emission of N_2_O of 242 ± 197 mg N_2_O‐N m^−2^ year^−1^. For the same six sites, Jauhiainen et al. (2023) inferred a mean soil CO_2_ removal factor of −23 ± 132 g CO_2_‐C m^−2^ year^−1^ using above and belowground litter production and decomposition rates in trenched plots, where their mean value is within the range of NEE totals we report for 2023 and 2024 but note their considerable uncertainty interval. Afforestation of a cutover Canadian peatland with Black spruce ( *Picea mariana* ) and paper birch ( *Betula papyrifera* ) had C balances 7 years after planting ranging from −72 ± 185 to −249 ± 191 g C m^−2^ year^−1^ depending on the fertiliser dose, and which were estimated using a combination of chamber fluxes and above and below ground productivity (Bravo et al. 2018). In the same study, the annual soil CH_4_ emission was estimated to be 1 ± 1 g CH_4_‐C m^−2^ year^−1^, comparable to values we found.

The results showed that afforestation halted annual C losses at the study site (Table 3). Using 2022 as the reference year and averaging the balances of 2023 and 2024, the mean change in NECB after fertilisation and afforestation is 172 g C m^−2^ year^−1^. The small CO_2_ sink was neutralised by DOC losses in 2023 and 2024, whereas CH_4_ did not contribute substantially to the NECB. The annual results also showed that fertilisation and afforestation caused negative radiative forcing (i.e., cooling effect) because of NEE uptake and the increase in surface albedo. Applying the same approach of using 2022 as the reference year and averaging the radiative balances of 2023 and 2024, there was a mean radiative forcing of −688 g CO_2_‐eq m^−2^ year^−1^. During 2022, the saplings were planted, and the emergence of wild vegetation had begun because of fertilisation, which suggests that the mean before and after fertilisation and afforestation changes in the NEE, NECB, and radiative balances are likely to be greater than the values we report. A partial and flawed comparison could be made by calculating annual totals between September 2021 and September 2022; however, the annual total of NEE, which dominates the balances, was largely unchanged at 138 ± 19 g CO_2_‐C m^−2^ year^−1^, but even for that time period the planting of saplings and the beginning of the emergence of wild vegetation in July 2022 affects this total as well. There were also large changes in ditch CH_4_ fluxes between 2021 and 2022 (Figure 7), possibly caused by management effects related to afforestation, which also highlights a flaw in using 2022 as the reference. In any case, the annual totals we found for 2022 are still comparable to the mean of annual emissions (excluding ditches) from active peat extraction sites in Nordic countries (mean ± 1 SD) of 190 ± 72 g CO_2_‐C m^−2^ year^−1^, 1.24 ± 1.82 g CH_4_‐C m^−2^ year^−1^ and 57 ± 70 mg N_2_O‐N m^−2^ year^−1^ (Maljanen et al. 2010).

Our study is only a snapshot of the early stages of afforestation, and it is of key interest whether the site will remain a sink of CO_2_ on short‐term and long‐term timescales. It is evident that the residual peat continues decomposing, as reflected in the small net annual uptake of CO_2_ in 2023 and 2024. We have not measured the change in the C content in the different C pools at the site, and thus we can only assume where exactly the sequestered C has been stored. The above and belowground wildly emerged plant biomass and litter were the likely C pools that increased within the ecosystem and caused the negative annual totals of NEE. This is supported by a study of ash fertilisation on a cutaway peatland, which found that the biomass production and C stock of the emerged vegetation were more important than the tree seedlings, where the aboveground biomass of emerged ground vegetation was up to two times that of the tree saplings (Huotari et al. 2009). The growth of pine saplings and the naturally emerged birches will likely increase the CO_2_ uptake significantly in the coming years at our site. Also, the gradually developing litter layer will act as a temporary C sink, since there was no litter on top of the peat at the time of the afforestation. Eventually, the balance between C litter input and C output by heterotrophic respiration will change such that the amount of C in litter will not increase anymore and therefore diminish NEE. As the tree stand and vegetation composition evolve at the site, there will be further changes in litter input and decomposition in the future. Actions taken by the landholder, such as the thinning of naturally emerged vegetation, will also result in variation in source‐sink behaviour.

The transition to Scots pine forest is also of key interest. The continued drainage of the site may compromise the peat C storage and lead to continual losses (Jurasinski et al. 2024). Despite this, nutrient‐poor boreal forestry‐drained peatlands have been shown to still accumulate C even when drained, where litter C inputs outpace peat losses (Lohila et al. 2011; Minkkinen et al. 2018; Ojanen et al. 2013), but in nutrient‐rich forests, the C losses from soil outpace accumulation due to higher rates of microbial peat decomposition in boreal peatlands (Korkiakoski et al. 2023; Meyer et al. 2013; Ojanen et al. 2013) and in temperate peatlands (Jovani‐Sancho et al. 2021). The calculated emission factors (EFs) for drained nutrient‐poor peatland forests in Finland indicate they are a net CO_2_ sink of −19 g CO_2_‐C m^−2^ year^−1^, a small CH_4_ sink of −0.1 CH_4_‐C m^−2^ year^−1^, and a N_2_O source of 70 mg N_2_O‐N m^−2^ year^−1^ (Minkkinen et al. 2020; Ojanen et al. 2019, 2020). However, although the EFs of CH_4_ and N_2_O of nutrient‐rich peatland forests are similar to nutrient‐poor peatland forests (Minkkinen et al. 2020; Ojanen et al. 2010), nutrient‐rich peatland forests are net CO_2_ sources of 66 g CO_2_‐C m^−2^ year^−1^ (Ojanen and Minkkinen 2019).

In the long term, over several tree stand rotations, the afforestation may not have a net cooling effect on the climate, if the loss of peat C in continued oxidation exceeds the input of C in litter to the soil (Ojanen et al. 2013), since the C sequestered in the tree stand will be exported in harvests and will be released rather quickly back to the atmosphere (Korkiakoski et al. 2019, 2023). When the situation is framed as a comparison of abandonment or afforestation of the cutaway peatlands, then afforestation appears to be a better alternative in terms of net climate impact. The NEE at our site was 144 ± 20 g C m^−2^ year^−1^ in 2022, before the fertilisation strongly affected the site vegetation. Emissions from abandoned peat extraction sites in more southern conditions are greater, where values of 285 g CO_2_‐C m^−2^ year^−1^ were determined in Estonia (Salm et al. 2012), 269 g CO_2_‐C m^−2^ year^−1^ in New Zealand (Rutledge et al. 2010), 88–399 g CO_2_‐C m^−2^ in summer (Waddington et al. 2002) and 173–259 g CO_2_‐C m^−2^ year^−1^ (Rankin et al. 2018) in Canada, and the aforementioned Nordic wide mean EFs of 190 ± 72 g CO_2_‐C m^−2^ year^−1^ (Maljanen et al. 2010).

### 4.2 Comparison to Other Land Use Conversion Options

The main alternative after‐use options of peat extraction are agriculture, solar or wind power farms, and restoration and rewetting (Karofeld et al. 2017; Laasasenaho et al. 2023; Räsänen et al. 2023). There are many options in agriculture which make it difficult to generalise, but a land use change to agriculture would likely result in higher emissions of CO_2_ and N_2_O. Nordic EFs for agriculture on peat soil are 488 g CO_2_‐C m^−2^ year^−1^, 0.16 g CH_4_‐C m^−2^ year^−1^, and 0.99 g N_2_O‐N m^−2^ year^−1^ (Maljanen et al. 2010), whereas EFs specific to Finland have been determined to be 777 g CO_2_‐C m^−2^ year^−1^, 0.75 g CH_4_‐C m^−2^ year^−1^, and 1.09 g N_2_O‐N m^−2^ year^−1^ (Ojanen et al. 2020). As far as we could ascertain, there are no published studies of GHG exchange where the land use conversion of peat extraction sites to solar and wind power farms has been conducted. Restoration/rewetting is, besides forestry, the other after‐use that can maintain the C storage function of peatlands long‐term. In ideal cases, restoration results in the return of a net C sink and a net climate cooling effect. The emissions of CH_4_ can pose an issue in the short term (e.g., Hemes et al. 2018) but despite this, in the long term, rewetting generally reduces climate warming effects (Günther et al. 2020). There can be novel and variable responses to rewetting (Kreyling et al. 2021), and it can take several years to decades or even centuries to lower the net warming impact (Ojanen et al. 2020; Wilson et al. 2016). For example, a restored cutaway peatland in Germany was still an annual source of CO_2_, CH_4_, and N_2_O of 20 g CO_2_‐C m^−2^ year^−1^, 6.5 g CH_4_‐C m^−2^ year^−1^, and 89 mg N_2_O‐N m^−2^ year^−1^, respectively, 18 years after restoration began (Schaller et al. 2022), but a restored cutaway peatland in Finland was estimated to be a growing season sink (not annually) of −136 ± 112 g CO_2_‐C m^−2^ 10 years after restoration and was not statistically different from the total of a pristine site of −106 ± 72 g CO_2_‐C m^−2^ (Soini et al. 2010). A study on restored Swiss cutaway peatlands found two sites aged 42 and 51 years after peat extraction had their growing season C‐sink function restored to −222 and −209 g CO_2_‐C m^−2^, respectively, whereas one younger site of 29 years since extraction was a net source of 40 g CO_2_‐C m^−2^, and where the differences in fluxes were due to age differences and *Sphagnum* cover (Samaritani et al. 2011).

### 4.3 Changes in Land Cover and Fluxes

Measured albedos transitioned from typical values observed in other studies of bare peat of 0.08 (Worrall et al. 2020) to those within the range of typical grasslands of 0.12–0.14 (Cescatti et al. 2012). Our results showed that the radiative forcing from changes in surface albedo, relative to the reference period prior to afforestation, made meaningful contributions to the radiative balance compared to the CO_2_‐eq GHG balance (Table 3) and reinforces that albedo effects should not be ignored when examining the net climate impact of land cover changes (Chen et al. 2024). We ignored the effect of snow albedos in our annual results because we do not expect the low vegetation and very sparse permanent canopy of the small pine trees to have a large impact compared to bare peat soil. The temporally variable snow cover length can have a large impact on annual RF that hides the effect of changes in vegetation cover, as we show in Supporting Information S1. We also note that there are uncertainties in the EESF/TH calculation stemming from the field of view of the pyranometer, which may not represent the full variability in albedo across the site, the prescribed/subjective reference scenario, which needs to be defined to calculate RF_Δ*α*_, and fundamentally from the conversion of an instantaneous RF in RF_Δ*α*_ to CO_2_‐eq in EESF/TH (Bright and Lund 2021).

Albedo changes with the forest transition will also be important for the radiative balance. For example, the albedo changes caused by boreal forestation of cultivated land can outweigh the C sequestration effects on net radiative forcing (Betts 2000; Jackson et al. 2008; Lohila et al. 2010). More radiation is absorbed by forests than by natural mire vegetation in summer because the forest canopy is darker (Harding and Pomeroy 1996). This heating effect may be partly compensated by enhanced evaporation in forests (Bala et al. 2007). There may be a small reduction in summer albedo as our site matures to a Scots pine forest, where mean mid‐summer albedos of mature Scots pine forests are around 0.11–0.12 (Lohila et al. 2010). In particular, winter albedos of forests tend to be much lower than low vegetation or bare soils because of lower snow cover and less direct radiation reflectance due to canopy scatter (Peräkylä et al. 2025). In our case, the RF_Δ*α*_ of long‐term forestation on former peat extraction sites would depend on the reference scenario chosen, whether that be abandonment (with the eventual return of vegetation cover) or conversion to another land use (e.g., restoration), and is an interesting area for future research.

Satellite imagery highlighted the vast changes in land cover over time at the study site (Figure 2). The changes in LAI and NDVI were largely caused by the emergence of wild plant species rather than the Scots pine saplings, given the planting density of 0.18–0.19 trees m^−2^ and the small size of the saplings due to their age. We found that changes in LAI were strongly related to changes in C fluxes. Another study also found good relationships between a different satellite LAI product (MODIS MCD15A3H.006) and GPP derived from global FLUXNET sites of *r* = −0.47 to −0.97 and slopes of −3.4, −2.2, and −1.8 $g\;{\mathrm{CO}}_{2}\text{‐}C\;m_{\mathrm{leaf}}^{-2}\;{\mathrm{day}}^{-1}$ were found for savannah, grassland, and evergreen broadleaf forest sites, respectively (Hoek van Dijke et al. 2020). The LAI‐GPP slope in our study was steeper (−4.04 $g\;{\mathrm{CO}}_{2}\text{‐}C\;m_{\mathrm{leaf}}^{-2}\;{\mathrm{day}}^{-1}$), which may be attributed to the rapid growth of vegetation on fertilised bare land.

Mean annual CH_4_ emissions varied little over the study period (Table 3). From our measurements, there appears to be a gradual yearly increase in strip FCH_4_ that may be related to land cover changes (Figure 7c); however, our unequal temporal sampling makes it difficult to conclusively state any trends. For ditch FCH_4_, there was a clear increase in emissions between the years 2021 and 2022 (Figure 7c). It is unclear why the ditch emissions increased in 2022. Ditch cleaning, which was performed at our site in 2022, was found to reduce ditch CH_4_ emissions in a post‐harvest forest where the cleaning likely resulted in a reduction of waterlogging in the ditch (Tong et al. 2022) but in another study the cleaning resulted in higher ditch emissions particularly where there was removal of moss coverage (Rissanen et al. 2023). The change in ditch CH_4_ emissions, therefore, likely depends upon the changes in hydrological conditions and plant coverage and requires further investigation. Ditches are well‐known hotspots of CH_4_ emissions (Hendriks et al. 2024; Minkkinen and Laine 2006; Peacock et al. 2021), and they may also be important emitters of N_2_O (Silverthorn et al. 2025). In our data, N_2_O emissions were higher from the ditches than from the strips. Along with the drainage water from the strips, nutrients are also drained to the ditches, and under anoxic conditions in the ditch, N_2_O will be formed from N compounds through denitrification (Webb et al. 2021). Annual N_2_O emissions appeared to trend downwards (Table 3). The decrease in N_2_O emissions may be related to the increase in vegetation cover since there would be greater N demand and uptake by plants, where N may otherwise be mineralised under barren conditions (Korkiakoski et al. 2019). However, it is difficult to conclusively state trends in N_2_O fluxes because of the low sampling frequency. N_2_O emissions are especially episodic, and a low temporal sampling frequency can under‐ or overestimate annual N_2_O flux totals by a large margin (Barton et al. 2015). Under‐sampling of winter and early spring N_2_O fluxes can bias annual totals since a high proportion of annual totals can come during this period, typically between a quarter and a half of emissions in drained boreal peatlands (Alm et al. 1999; Maljanen et al. 2003; Rautakoski et al. 2024; Regina et al. 2004).

The losses of C via DOC leaching are comparable to the IPCC default EF of 12 g C m^−2^ year^−1^ (95% CI: 0.07–0.19 g C m^−2^ year^−1^) for drained boreal organic soils (IPCC 2014). It is difficult to determine whether land cover changes have already affected DOC leaching at our site. The increase in vegetation cover may have improved soil stability and therefore decreased erosion of the peat and transport of particulate organic carbon (Dhillon and Inamdar 2014). This effect may be weak at present and could strengthen with forest development. Previous studies have found that more DOC is exported from cutover peatlands than from cutover sites restored with their natural peatland function, where there was also a clear link between precipitation amount and greater DOC export (Waddington et al. 2008). Seasonal TOC losses from weeks 18 to 44, of which the authors report 94% was DOC, from a former cutaway in Finland vegetated with reed canary grass had a mean annual value of 6.4 g C m^−2^ year^−1^ between 3 and 6 years after the cessation of peat extraction (Hyvönen et al. 2013). This speculatively suggests that DOC losses may decline with time, but as the site remains drained and soil layers remain unsaturated, most probably, DOC leaching from soil layers remains higher than in pristine and restored ones. Although DOC fluxes are ordinarily considered low and often ignored in temperate peatland settings (e.g., Boonman et al. (2024), Tiemeyer et al. (2024)), the C lost via DOC effectively offsets the C uptake via NEE in 2023 and 2024 (Table 3) and should be taken into account in C budget estimates in future studies.

Changes in Bowen ratios at our site were difficult to directly link to changes in land cover and seemed to be driven by water availability. We anticipated that the increased vegetation cover and higher albedos compared to bare peat soil should theoretically reduce H (sensible heat) uptake and increase LE (latent heat) because of higher transpiration. For example, Bowen ratios in boreal forest were between 1.0 and 2.7 in the season after clearcutting and were mostly between 0.6 and 1.0 in the following year when there was higher coverage of ground vegetation (Korkiakoski et al. 2019). Future analyses could try and account for water availability when analysing the partitioning of energy fluxes to search for a more insightful relationship.

## 5 Conclusions

We present the first study with continuous monitoring of CO_2_ fluxes from a newly afforested and fertilised cutaway peatland. Addressing our aims specifically, we found that (1) afforestation, which included wood ash fertilisation prior to the planting of Scots pine saplings, led to a large change in landcover where natural vegetation quickly emerged in the site after fertilisation as corroborated by satellite imagery indexes, (2) afforestation‐related fertilisation caused a greater exchange of CO_2_, with increases in both Reco and GPP. We found that afforestation‐related fertilisation quickly turned our study site from a source of CO_2_ in the first year to a sink in the next two study years, and the increases in surface albedo resulted in negative radiative forcings, and (3) the site was a small annual source of DOC, CH_4_, and N_2_O over the study period. Afforestation‐related fertilisation caused a large mean change in the NECB of 172 g C m^−2^ year^−1^, and our results indicate that net annual C losses have halted at our site. Additionally, we found that afforestation‐related fertilisation caused a large mean reduction in radiative balance of −688 g CO_2_‐eq m^−2^ year^−1^ over a 100‐year time horizon because of the changes in NEE and surface albedo. Since cutaway peatlands can be persistent sources of C for decades if abandoned, our results show that the CO_2_ uptake by wild vegetation emerging during the afforestation process compensates for C losses by peat oxidation and DOC losses within 2 years. On a longer timescale, it is uncertain whether afforestation of peat extraction sites will have a net cooling climate effect because of peat oxidation due to drainage, C losses from forest harvests, and changes in surface albedo with forest development. We anticipate the results of the study will aid and guide land use change and policy decisions on the after‐use of northern peat extraction areas. A key remaining question is whether the site will remain a sink of CO_2_ in the short term and the long term. Measurements continue at the site, and the situation will be monitored for a better understanding of CO_2_ source/sink behaviour and changing surface albedo as the site develops. It was difficult to conclusively state trends in the chamber fluxes of CH_4_ and N_2_O, as their annual totals had large uncertainties because these fluxes had high spatial and temporal variation. In the future, the fluxes of CH_4_ and N_2_O will also be measured with EC to provide near‐continuous measurements while integrating spatial variability.

## Author Contributions

**Alexander J. V. Buzacott:** data curation, formal analysis, investigation, methodology, visualization, writing – original draft, writing – review and editing. **Kari Laasasenaho:** conceptualization, funding acquisition, project administration, writing – review and editing. **Risto Lauhanen:** conceptualization, funding acquisition, project administration, writing – review and editing. **Kari Minkkinen:** conceptualization, funding acquisition, methodology, project administration, writing – review and editing. **Paavo Ojanen:** conceptualization, funding acquisition, methodology, project administration, writing – review and editing. **Gopal Adhikari:** data curation, formal analysis, investigation, writing – review and editing. **Liisa Jokelainen:** data curation, formal analysis, investigation, writing – review and editing. **Lassi Päkkilä:** data curation, formal analysis, investigation, writing – review and editing. **Hannu Marttila:** conceptualization, data curation, formal analysis, funding acquisition, project administration, writing – review and editing. **Annalea Lohila:** conceptualization, funding acquisition, methodology, project administration, writing – original draft, writing – review and editing.

## Funding

This work was supported by Maa‐ja Metsätalousministeriö; Centre for Economic Development, Transport and the Environment, Central Finland (2021/901561/09, 2021/901901/09); and Research Council of Finland (345510).

## Conflicts of Interest

The authors declare no conflicts of interest.

## Supporting information


**Supporting Information S1:** gcb70644‐sup‐0001‐Supinfo1.pdf.

**Supporting Information S2:** gcb70644‐sup‐0002‐Supinfo2.pdf.

**Supporting Information S3:** gcb70644‐sup‐0003‐Supinfo3.pdf.

## Data Availability

The data and code that support the findings of this study are openly available in Dryad at https://doi.org/10.5061/dryad.2fqz61339 and Zenodo at https://zenodo.org/records/17697089. Sentinel‐2 satellite data were obtained from Google Earth Engine at https://developers.google.com/earth‐engine/datasets/catalog/COPERNICUS_S2_SR_HARMONIZED. Finnish Meteorological Institute (FMI) meteorological data were obtained at https://en.ilmatieteenlaitos.fi/open‐data. Finnish Environment Institute (SYKE) catchment‐specific discharge data were obtained at https://wwwp2.ymparisto.fi/scripts/kirjaudu.asp.

## Appendix A EC Processing and Gapfilling

The LI‐7200RS inlet line was vertically and horizontally separated from the midpoint of the sonic anemometer by 12 and 3 cm, respectively. Sample air was pumped at 12 L min^−1^ along a 1 m long heated tube of 4.57 mm in diameter, which included an inline Swagelock 2 μm filter (Swagelock, Solon OH, USA). EC data were collected at a 10 Hz frequency. EC fluxes were processed using EddyUH (Mammarella et al. 2016) using standard flux processing techniques and corrections (Sabbatini et al. 2018). Raw data were despiked using median absolute deviation (MAD) approach method of Mauder et al. (2013), where the *q* threshold value was set to 7. The LI‐7200RS internally corrects for water‐induced density fluctuations and further corrections are not necessary. The crosswind correction of Liu et al. (2001) was applied to sonic anemometer temperature data. The two‐dimensional coordinate rotation method was used for to account for potential sonic misalignment (Wilczak et al. 2001). Time lags were accounted for using the covariance maximisation method, which were determined experimentally for CO_2_ and H_2_O following Mammarella et al. (2009). A timelag of 0.3 s was determined for CO_2_, and the timelag of H_2_O was a function of relative humidity due to increased sorption of water in the inlet line at higher relative humidity, where typical lag times of 0.3 s were found for 30%–40% relative humidity and were more than 1 s for relative humidity greater than 80%. Covariances were calculated between the vertical wind speed and scalars of interest using an averaging period of 30‐min, where the turbulent fluctuations were detrended by block averaging. Corrections for low and high frequency losses due to the finite averaging period and limited frequency response, respectively, were applied. The theoretical corrections of Moncrieff et al. (2004) were applied to correct for low frequency loss, and the experimental approach of Mammarella et al. (2009) was used to correct for high frequency losses where the low‐pass filter time constant was estimated for each gas.

Half‐hourly fluxes were filtered for quality according to the Foken et al. (2004) quality system and fluxes with an overall quality flag value of 4 or below were accepted for further analysis. Data were then screened by absolute realistic limits for the site and were despiked with the median absolute deviation filter after Papale et al. (2006). The net ecosystem exchange (NEE) of CO_2_ was calculated by adding the single point storage estimate to the half‐hourly flux of CO_2_ (Aubinet et al. 2012). Poorly turbulent nighttime records of NEE were removed using a friction velocity (*u**) threshold determined using the moving point test (Papale et al. 2006). Thresholds of *u** were determined annually and ranged from 0.13 to 0.16 m s^−1^.

The ML gapfilling of NEE involved introducing artificial gaps to the dataset, creating training, testing and validation sets, and followed by hyperparameter tuning of the XGBoost models. Artificial gaps were first introduced into the dataset using the method described in Irvin et al. (2021). The method aims to preserve gap distributions to ensure that gaps of all lengths are tested across the training, validation, and testing sets. One held‐out test set was created to assess the ensemble mean prediction, followed by creating 10 pairs of training and validation sets with their own samples of artificial gaps (Moffat et al. 2007). The XGBoost model hyperparameters were tuned using 5‐fold cross‐validation on each of the 10 training sets. The predictors included for training were TA, PAR, vapour pressure deficit (VPD), and the fuzzy temporal variables yearly sine and cosine, hourly sine and cosine, and day of year delta. After the models were trained, the mean values of the ensemble of 10 tuned models were used to fill gaps in the NEE timeseries. The uncertainty of individual predictions was taken from the variance of the model ensemble. Annual fluxes were calculated using the cumulative sum of the observed and gapfilled data. Annual uncertainty was estimated using the variance of the annual totals from the 10 tuned models plus the variance due to uncertain *u** threshold. The same procedure was applied as FLUXNET for annual uncertainty derived from the *u** threshold, where 40 quantiles of the *u** threshold linearly spaced between 0.0125 and 0.9875 were used to filter the timeseries (Pastorello et al. 2020) and which were then propagated through the ML gapfilling procedure.
