# Supplementary material for: Afforestation‐Related Fertilisation Quickly Turns Barren Cutaway Peatland Into a Carbon Dioxide Sink
Source: Glob Chang Biol. 2025 Dec 17;31(12):e70644. doi: 10.1111/gcb.70644 (PMC12710595; doi:10.1111/gcb.70644)
Supplement: Supplementary file 1 — Supporting Information S1: gcb70644‐sup‐0001‐Supinfo1.pdf. [file GCB-31-e70644-s003.pdf]

Supporting information S1 to:

Afforestation-related fertilisation quickly turns barren cutaway peatland into a carbon dioxide sink

Alexander J.V. Buzacott[1], Kari Laasasenaho[2], Risto Lauhanen[2], Kari Minkkinen[3], Paavo Ojanen[3,4], Gopal Adhikari[3], Liisa Jokelainen[3], Lassi Päkkilä[5], Hannu Marttila[5], Annalea Lohila[1,6]

[1]Institute for Atmospheric and Earth System Research/Physics, University of Helsinki, Pietari Kalmin katu 5, Helsinki, 00560, Finland
[2]Seinäjoki University of Applied Sciences, P.O. Box 412 / Frami F, Kampusranta 11, FI-60101 Seinäjoki, Finland
[3]Department of Forest Sciences, University of Helsinki, Latokartanonkaari 9, 00790 Helsinki, Finland
[4]Natural Resources Institute Finland, Latokartanonkaari 9, 00790 Helsinki, Finland
[5]Water, Energy and Environmental Engineering Research Unit, Faculty of Technology, P.O. Box 4300, FI-90014 University of Oulu, Finland
[6]Climate System Research, Finnish Meteorological Institute, Erik Palménin aukio 1, Helsinki, 00560, Finland

Correspondence to: Alexander J.V. Buzacott (alexander.buzacott@helsinki.fi)

Contents:

- **Figure S1.1.** Mean daily timeseries of incoming shortwave radiation at the top of atmosphere (SW_{TOA}), incoming shortwave radiation measured at the site surface (SW_{in}) and mean daily surface albedo (ALB) calculated from measurements at Naarasneva.

- **Figure S1.2.** Timeseries of monthly mean incoming shortwave radiation at the top of atmosphere (SW_{TOA}) and incoming shortwave radiation measured at the site surface (SW_{in}), and monthly mean surface albedo (ALB) calculated from mean daily measurements of albedo at the study. The bottom plot shows an enhanced view of albedo for the warmer season months.

- **Table S1.1.** Monthly surface albedos (α) at Naarasneva. Albedos were calculated as the ratio of outgoing and incoming shortwave radiation. Monthly values were calculated from daily means of albedo.

- **Table S1.2.** Reference albedos (α_{ref}) selected for Naarasneva.

- **Table S1.3.** Annual radiative forcing ($RF_{\Delta\alpha}$) and CO_2-equivalent metrics.

In this supplement, we expand on the approach to calculate the radiative forcing (RF) of albedo (α) change at Naarasneva and the CO_2-equivalent impact.

To calculate the RF due to albedo change ($\Delta\alpha$), we used the parameterisation after Bright & O'Halloran (2019):

$$\mathrm{RF}_{\Delta\alpha}(t) = \frac{1}{12}\sum_{m=1}^{m=12} -\mathrm{SW}_{\mathrm{in}(m,t)}\sqrt{T_{a(m,t)}}\Delta\alpha_{(m,t)} \tag{S1.1}$$

where $\mathrm{RF}_{\Delta\alpha}$ is the annual RF due to a change in surface albedo at year t and the units are W m^{-2}, $\mathrm{SW}_{\mathrm{in}}$ is incoming shortwave radiation measured at the surface at year t and month m, T_a is the upward atmospheric transmittance and $\Delta\alpha$ is the change in surface albedo. Assuming downward and upward transmittances to be equal, T_a was approximated as the ratio of $\mathrm{SW}_{\mathrm{in}}/\mathrm{SW}_{\mathrm{TOA}}$ where $\mathrm{SW}_{\mathrm{TOA}}$ is incoming shortwave radiation at the top of atmosphere (TOA). $\mathrm{SW}_{\mathrm{in}}$ was derived from monthly mean midday measurements (between 10:00 and 16:00) at the site surface, and monthly mean $\mathrm{SW}_{\mathrm{TOA}}$ was calculated from daily approximations after Duffie & Beckman (2013):

$$\mathrm{SW}_{\mathrm{TOA}} = \frac{S_0}{\pi}\left(1 + 0.033\cos\left(\frac{\pi}{180}\frac{360\mathrm{DOY}}{365}\right)\right)(\cos\phi\cos\delta\sin\omega_s + \omega_s\sin\phi\sin\delta) \tag{S1.2}$$

where S_0 is the solar constant (1367 W m^{-2}), DOY is the decimal day of year, ϕ is the latitude of the site, δ is the solar declination angle, and ω_s is the solar zenith angle (latitude and angles expressed in radians). The timeseries of $\mathrm{SW}_{\mathrm{TOA}}$ is displayed in Figures S1.1 and S1.2.

The timeseries of daily and monthly variables are shown in Figure S1.1 and Figure S1.2, which include $\mathrm{SW}_{\mathrm{TOA}}$, $\mathrm{SW}_{\mathrm{in}}$ measured at Naarasneva with a pyranometer, and surface albedo which is the ratio of outgoing shortwave radiation ($\mathrm{SW}_{\mathrm{out}}$) to $\mathrm{SW}_{\mathrm{in}}$ measured with the pyranometer at Naarasneva. As above, only half-hourly measurements between 10:00 and 16:00 were used for aggregation of albedo, and measurements of $\mathrm{SW}_{\mathrm{in}}$ and $\mathrm{SW}_{\mathrm{out}}$ were used to calculate albedo if they were at least 2 W m^{-2}.

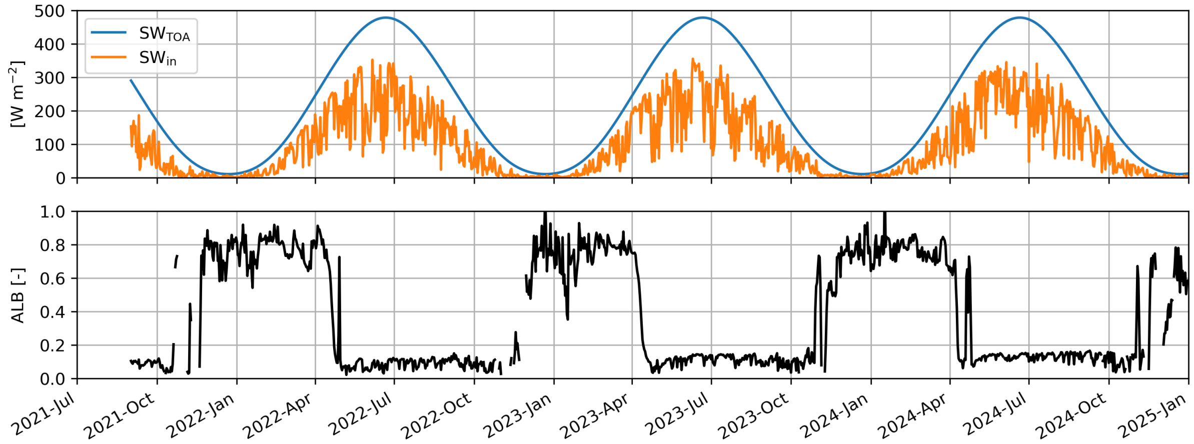

Figure S1.1. Mean daily timeseries of incoming shortwave radiation at the top of atmosphere ($\mathrm{SW}_{\mathrm{TOA}}$), incoming shortwave radiation measured at the site surface ($\mathrm{SW}_{\mathrm{in}}$) and mean daily surface albedo (ALB) calculated from measurements at Naarasneva.

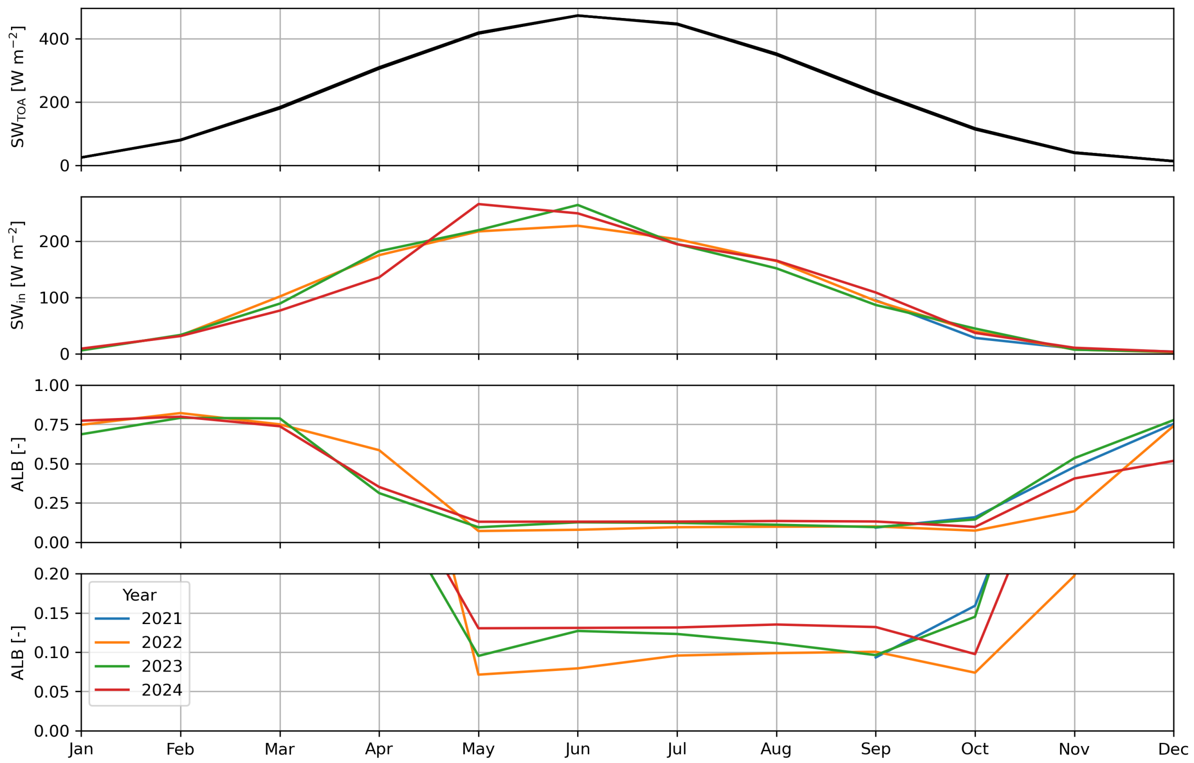

Figure S1.2. Timeseries of monthly mean incoming shortwave radiation at the top of atmosphere (SW_{TOA}) and incoming shortwave radiation measured at the site surface (SW_{in}), and monthly mean surface albedo (ALB) calculated from mean daily measurements of albedo at the study. The bottom plot shows an enhanced view of albedo for the warmer season months.

A reference albedo situation needs to be defined to calculate $\Delta\alpha$. In our case, the reference situation represents the albedo if no land use change had occurred, i.e. the peat would still be bare. Reference albedos were split between when the peat would be bare and when there would typically be snow cover. For the reference for the bare peat period (May to September), we used measurements from 2021 and 2022 that characterise typical albedos of bare peat. From Table S1.1, we can see values range from 0.07 to 0.10 during that time, with a typical value of 0.08 that seems to characterise the bare peat and which agrees with prior studies (e.g. Worral et al. (2020)). Hence, a reference value of 0.08 was chosen to represent the surface albedo bare peat for the months after fertilisation/afforestation effects on land cover begin. The bare peat reference values are constant in time, since without fertilisation or other intervention (e.g. ditch blocking), peat extraction areas typically remain barren for long periods of time (Taylor & Price, 2015; Waddington et al., 2002). For months that were typically affected by snow cover (October to April), we used month means across all years to serve as the reference for those months. These months were selected based on their higher means and/or standard deviations which indicates snow presence (Table S1.1, Figure S1.2).

The reference albedo values for Naarasneva before afforestation are presented in Table S1.2. Long-term bare-peat surface albedos can be obtained from satellite data, such as the MODIS satellite, however the resolution of the product is quite coarse at 500 m which results in only 1 pixel covering the area of interest at our site and hence was not used.

Table S1.1. Monthly surface albedos (α) at Naarasneva. Albedos were calculated as the ratio of outgoing and incoming shortwave radiation. Monthly values were calculated from daily means of albedo.

		Monthly α [-]				
**Year**	**Month**	**Mean**	**SD**	**Q_{25}**	**Q_{50}**	**Q_{75}**
2021	9	0.09	0.01	0.09	0.10	0.10
2021	10	0.16	0.22	0.05	0.07	0.11
2021	11	0.48	0.34	0.07	0.62	0.78
2021	12	0.75	0.08	0.70	0.79	0.81
2022	1	0.75	0.08	0.68	0.75	0.81
2022	2	0.82	0.04	0.81	0.82	0.84
2022	3	0.75	0.06	0.70	0.74	0.81
2022	4	0.59	0.29	0.32	0.71	0.81
2022	5	0.07	0.03	0.05	0.07	0.08
2022	6	0.08	0.03	0.05	0.08	0.10
2022	7	0.10	0.02	0.08	0.10	0.11
2022	8	0.10	0.02	0.09	0.10	0.11
2022	9	0.10	0.03	0.08	0.11	0.12
2022	10	0.07	0.03	0.05	0.08	0.10
2022	11	0.20	0.16	0.11	0.15	0.21
2022	12	0.74	0.14	0.67	0.75	0.82
2023	1	0.69	0.14	0.62	0.68	0.79
2023	2	0.79	0.05	0.77	0.79	0.84
2023	3	0.79	0.03	0.77	0.78	0.80
2023	4	0.31	0.28	0.10	0.17	0.60
2023	5	0.10	0.03	0.07	0.10	0.12
2023	6	0.13	0.02	0.12	0.13	0.14
2023	7	0.12	0.02	0.11	0.13	0.14
2023	8	0.11	0.02	0.10	0.11	0.13
2023	9	0.10	0.03	0.07	0.10	0.12
2023	10	0.15	0.13	0.09	0.11	0.13
2023	11	0.54	0.21	0.51	0.53	0.72
2023	12	0.78	0.06	0.74	0.76	0.81
2024	1	0.77	0.10	0.71	0.77	0.81
2024	2	0.80	0.04	0.77	0.80	0.83
2024	3	0.74	0.05	0.70	0.73	0.77
2024	4	0.35	0.27	0.10	0.28	0.65
2024	5	0.13	0.01	0.12	0.13	0.14
2024	6	0.13	0.01	0.12	0.13	0.15
2024	7	0.13	0.02	0.12	0.13	0.14
2024	8	0.14	0.02	0.12	0.14	0.15
2024	9	0.13	0.03	0.12	0.14	0.15
2024	10	0.10	0.04	0.07	0.10	0.13
2024	11	0.41	0.27	0.13	0.38	0.66
2024	12	0.52	0.16	0.40	0.56	0.63

Table S1.2. Reference albedos (α_{ref}) selected for Naarasneva.

Month	α_{ref}	Source
1	0.74 ± 0.11	Jan mean and SD 2022-2024
2	0.80 ± 0.05	Feb mean and SD 2022-2024
3	0.76 ± 0.06	Mar mean and SD 2022-2024
4	0.42 ± 0.30	Apr mean and SD 2022-2024
5	0.07 ± 0.03	2022-05
6	0.08 ± 0.03	2022-06
7	0.08 ± 0.03	2022-06
8	0.08 ± 0.03	2022-06
9	0.09 ± 0.01	2021-09
10	0.12 ± 0.13	Oct mean and SD 2021-2024
11	0.43 ± 0.28	Nov mean and SD 2021-2024
12	0.70 ± 0.15	Dec mean and SD 2021-2024

To compare the impact of albedo change with GHGs, we also present a CO_2-equivalent metric for albedo change similar to previous studies (Betts, 2000; Bright et al., 2015; Bright & Lund, 2021; Carrer et al., 2018):

$$\mathrm{EESF/TH} = \frac{\mathrm{RF}_{\Delta\alpha}}{k_{\mathrm{CO_2}} A_{\mathrm{E}} \mathrm{AF}} \frac{1}{\mathrm{TH}} \tag{S1.3}$$

where EESF / TH is the CO_2 emissions equivalent of shortwave forcing (EESF) effect of a change in albedo divided by a time horizon (TH) to give emissions in kg CO_2-eq m^{-2} yr^{-1}, k_{CO_2} is the global mean radiative efficiency of CO_2 (1.76×10^{-15} W m^{-2} kg^{-1}), A_E is the surface area of earth (5.101×10^{14} m^2), AF is the average airborne fraction of CO_2 and represents the proportion of anthropogenic emissions of CO_2 that remains in the atmosphere after a set time period. We used a TH of 100-years such that it can be compared easily with the other GHGs in the study, as described in the main article. Since a 100-year TH was chosen, the AF parameter was set to 0.48 which is the remaining fraction of CO_2 given by the Bern carbon cycle model (Joos et al., 2001) when integrating over the 100-year TH (Carrer et al., 2018).

The annual uncertainty of metrics was calculated as the sum of aggregated uncertainty from the variance of observed albedos for a month (Table S1.1) and from the variance of the reference albedo (Table S1.2), both which were propagated through Equation S1.1 and Equation S1.3. The uncertainties were added in quadrature, and the total uncertainty is presented as the 95% confidence interval.

The results of $RF_{\Delta\alpha}$ and the CO_2-equivalent metrics are shown in Table S1.3. The annual mean $RF_{\Delta\alpha}$ was calculated to be −2.1 ± 4.3, −0.8 ± 4.4, and −2.5 ± 3.3 W m^{-2} for 2022, 2023, and 2024, respectively. In the case of annual means, there is interannual variation in $RF_{\Delta\alpha}$ (and therefore EESF / TH and GWP_{100}) because of the increase in surface albedo due to vegetation increase at Naarasneva overtime and the interannual variation in snow cover length. For example, the year 2023 had lower winter and spring albedos (Table S1.2, Figure S1.3) which has offset the increase in summer albedo compared to the year 2022.

Since we do not expect the vegetation present at the study site to substantially change the snow cover and snow reflectance characteristics compared to bare peat yet, and because we are mainly interested in the effect of increased vegetation cover compared to bare peat, we removed the effect of snow albedo by setting $\Delta\alpha=0$ for months that typically had complete or partial snow cover (October to April). These results show a clear year-on-year decrease in $RF_{\Delta\alpha}$ of −0.2 ± 1.0, −1.6 ± 1.0, and −2.9 ± 1.1 W m^{-2} for 2022, 2023, and 2024, respectively. In terms of CO_2 equivalence, the EESF / TH results show the change in $RF_{\Delta\alpha}$ corresponds to net equivalent uptake of CO_2 of −4 ± 24, −38 ± 24, and −67 ± 25 g CO_2 m^{-2} yr^{-1} for 2022, 2023, and 2024, respectively (Table S1.3) due to the increase in surface albedo from increased vegetation ground cover..

Table S1.3. Annual radiative forcing due to the change in surface albedo ($RF_{\Delta\alpha}$) and carbon dioxide emissions equivalent of shortwave radiation forcing divided by a 100-year time horizon (EESF / TH). Presented are the annual estimates that use $\Delta\alpha$ for all months and where we set $\Delta\alpha=0$ for the months that typically have snow (October to April).

	Annual		Annual (snow $\Delta\alpha=0$)	
**Year**	**$RF_{\Delta\alpha}$**	**EESF / TH**	**$RF_{\Delta\alpha}$**	**EESF / TH**
	[W m^{-2}]	[g CO_2 m^{-2} yr^{-1}]	[W m^{-2}]	[g CO_2 m^{-2} yr^{-1}]
**2022**	−2.1 ± 4.3	−49 ± 99	−0.2 ± 1.0	−4 ± 24
**2023**	−0.8 ± 4.4	−19 ± 103	−1.6 ± 1.0	−38 + 24
**2024**	−2.5 ± 3.3	−59 ± 77	−2.9 ± 1.1	−67 ± 25
