# Supplementary material for: Afforestation‐Related Fertilisation Quickly Turns Barren Cutaway Peatland Into a Carbon Dioxide Sink
Source: Glob Chang Biol. 2025 Dec 17;31(12):e70644. doi: 10.1111/gcb.70644 (PMC12710595; doi:10.1111/gcb.70644)
Supplement: Supplementary file 2 — Supporting Information S2: gcb70644‐sup‐0002‐Supinfo2.pdf. [file GCB-31-e70644-s001.pdf]

Supporting information S2 to:

Afforestation-related fertilisation quickly turns barren cutaway peatland into a carbon dioxide sink

Alexander J.V. Buzacott[1], Kari Laasasenaho[2], Risto Lauhanen[2], Kari Minkkinen[3], Paavo Ojanen[3,4], Gopal Adhikari[3], Liisa Jokelainen[3], Lassi Päkkilä[5], Hannu Marttila[5], Annalea Lohila[1,6]

[1]Institute for Atmospheric and Earth System Research/Physics, University of Helsinki, Pietari Kalmin katu 5, Helsinki, 00560, Finland
[2]Seinäjoki University of Applied Sciences, P.O. Box 412 / Frami F, Kampusranta 11, FI-60101 Seinäjoki, Finland
[3]Department of Forest Sciences, University of Helsinki, Latokartanonkaari 9, 00790 Helsinki, Finland
[4]Natural Resources Institute Finland, Latokartanonkaari 9, 00790 Helsinki, Finland
[5]Water, Energy and Environmental Engineering Research Unit, Faculty of Technology, P.O. Box 4300, FI-90014 University of Oulu, Finland
[6]Climate System Research, Finnish Meteorological Institute, Erik Palménin aukio 1, Helsinki, 00560, Finland

Correspondence to: Alexander J.V. Buzacott (alexander.buzacott@helsinki.fi)

Contents:

- **Figure S2.1.** Comparison of field observed and Sentinel-2 satellite derived leaf area index (LAI). Each box is generated from around 80 samples spread across the study site. The points for the satellite data are the mean of the satellite capture and the shading is the 95% confidence interval.
- **Figure S2.2.** Monthly median albedo calculated from observed incoming and outgoing shortwave radiation at Naarasneva. Timesteps between 10:00 to 16:00 were used to calculate the medians and the uncertainty interval is the interquartile range.
- **Figure S2.3.** Diurnal plots of sensible heat (H), latent heat (LE), Bowen ratio (β), net radiation (NETRAD), and ground heat flux (G). The points are monthly median values for each half-hour period.
- **Figure S2.4.** Boxplots including the values beyond the boxplot whiskers of (A) methane flux (FCH_4) and (B) nitrous oxide flux (FN_2O) and (c) dissolved organic carbon (DOC) concentrations. The number of values (*n*) used is printed below each box.
- **Figure S2.5.** Timeseries of methane (FCH_4) and nitrous oxide fluxes (FN_2O) measured from strips (left column) and ditches (right column) by the manual chamber method at Naarasneva.
- **Figure S2.6.** Timeseries of dissolved organic carbon (DOC) concentrations measured from samples taken at Naarasneva.

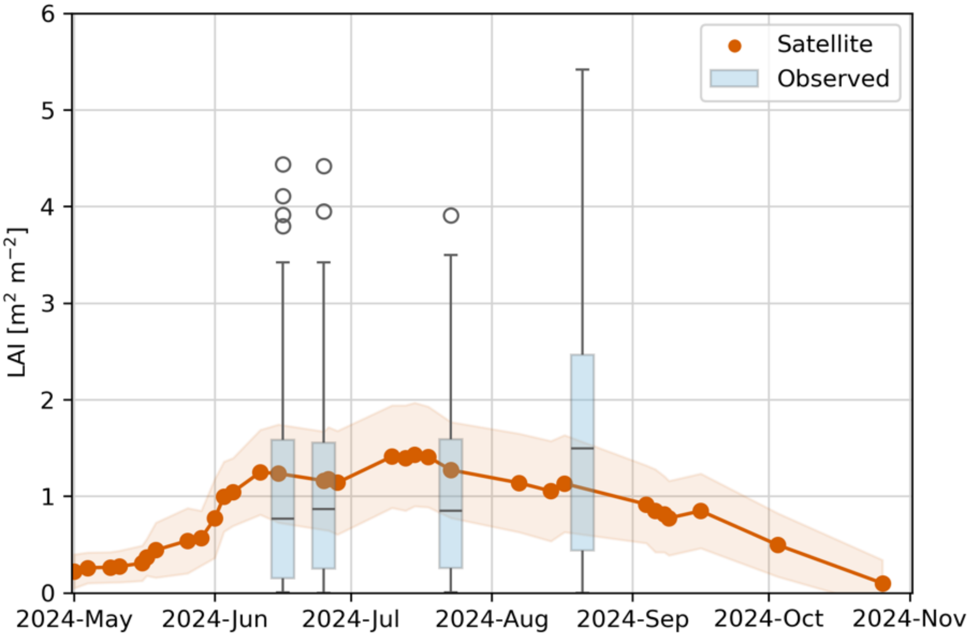

Figure S2.1. Comparison of field observed and Sentinel-2 satellite derived leaf area index (LAI). Each box is generated from around 80 samples spread across the study site. The points for the satellite data are the mean of the satellite capture and the shading is the 95% confidence interval.

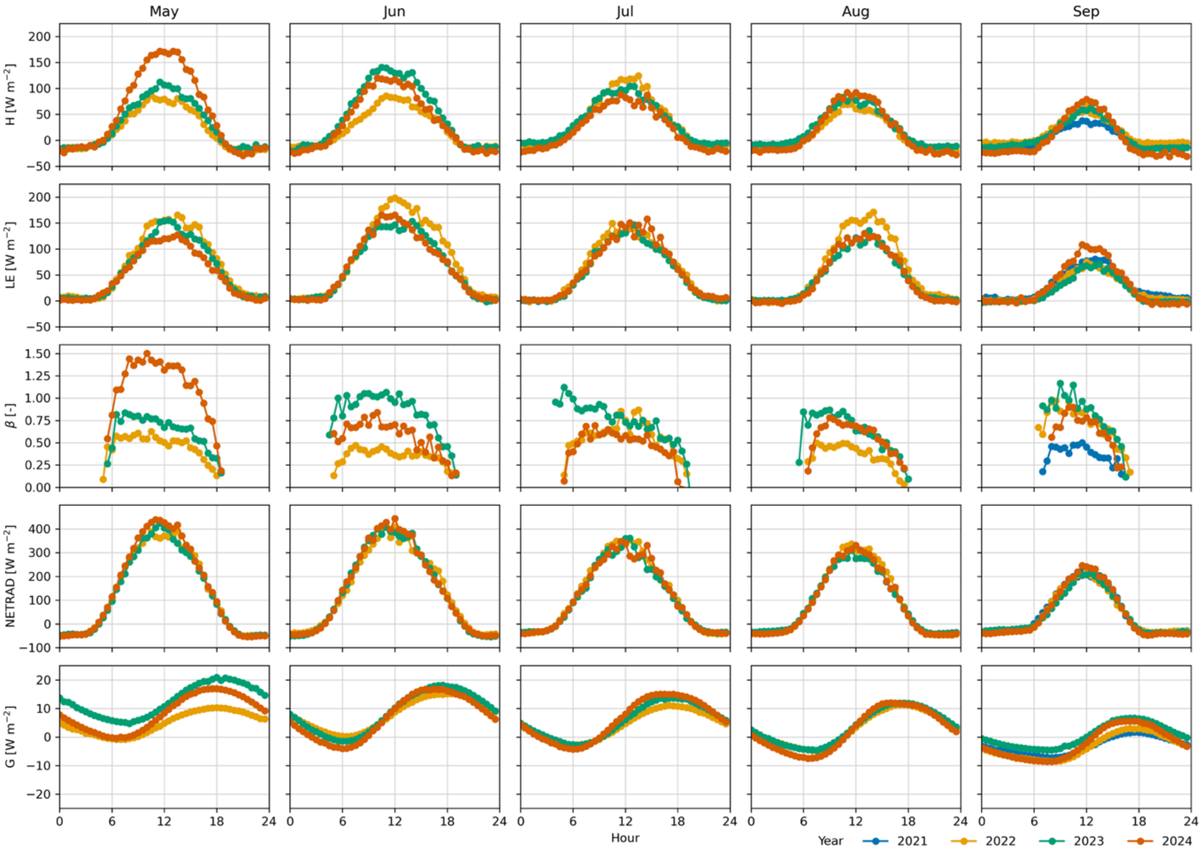

Figure S2.2. Diurnal plots of sensible heat (H), latent heat (LE), Bowen ratio (β), net radiation (NETRAD), and ground heat flux (G). The points are monthly median values for each half-hour period.

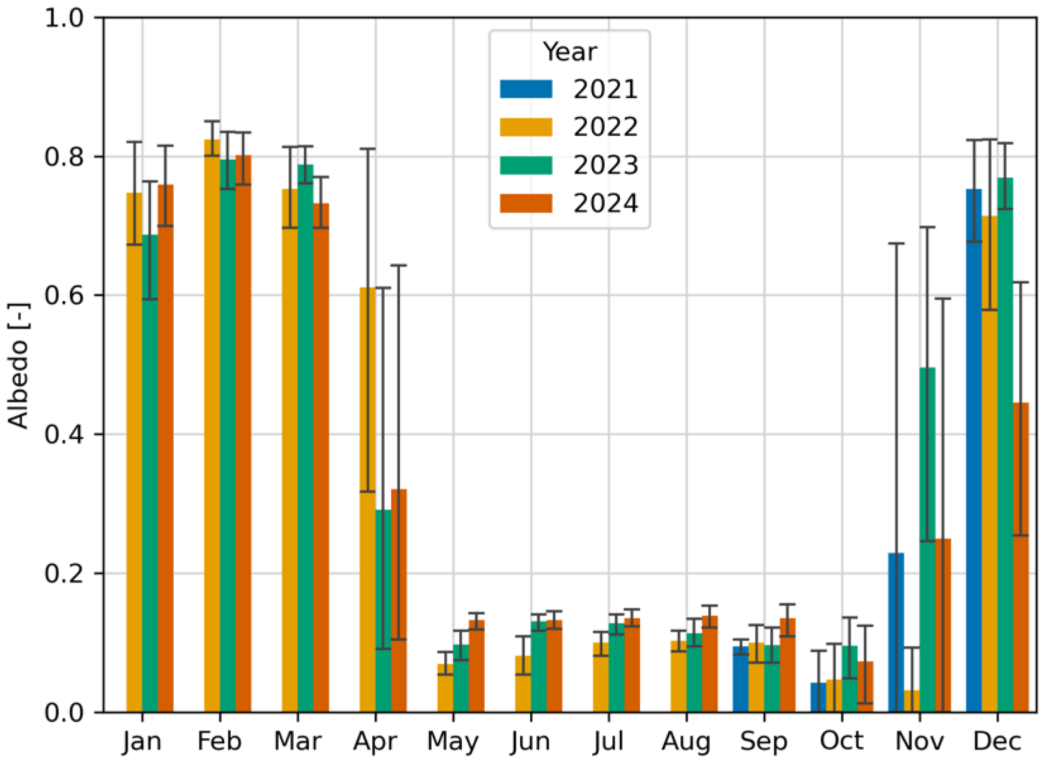

Figure S2.3. Monthly median albedo calculated from observed incoming and outgoing shortwave radiation at Naarasneva. Timesteps between 10:00 to 16:00 were used to calculate the medians and the uncertainty interval is the interquartile range.

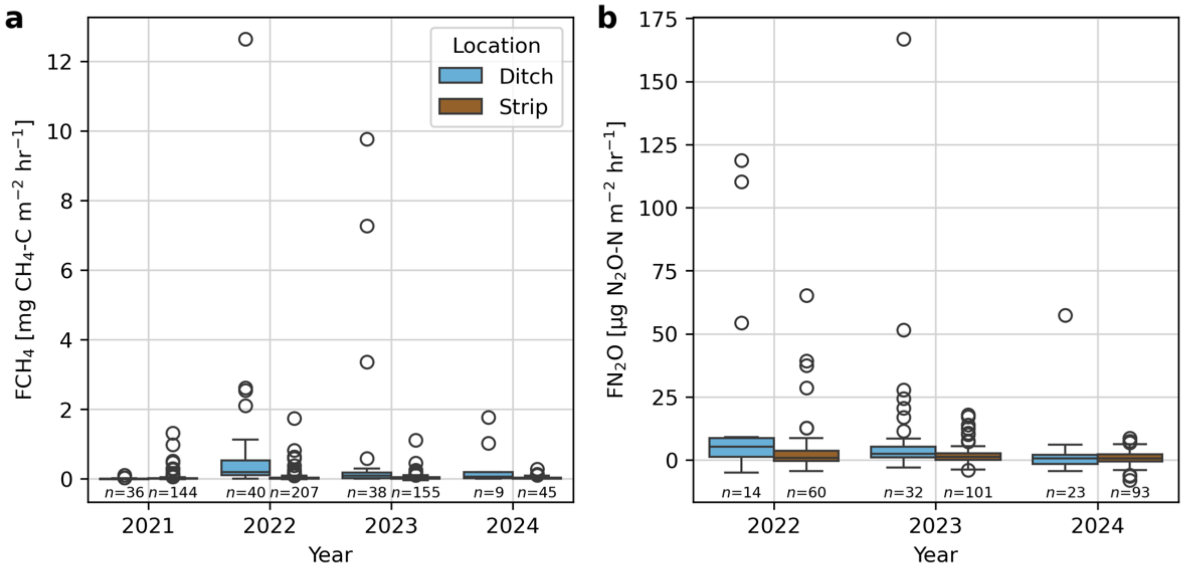

Figure S2.4. Boxplots including the values beyond the boxplot whiskers of (A) methane flux (FCH_4) and (B) nitrous oxide flux (FN_2O) and (c) dissolved organic carbon (DOC) concentrations. The number of values (*n*) used is printed below each box.

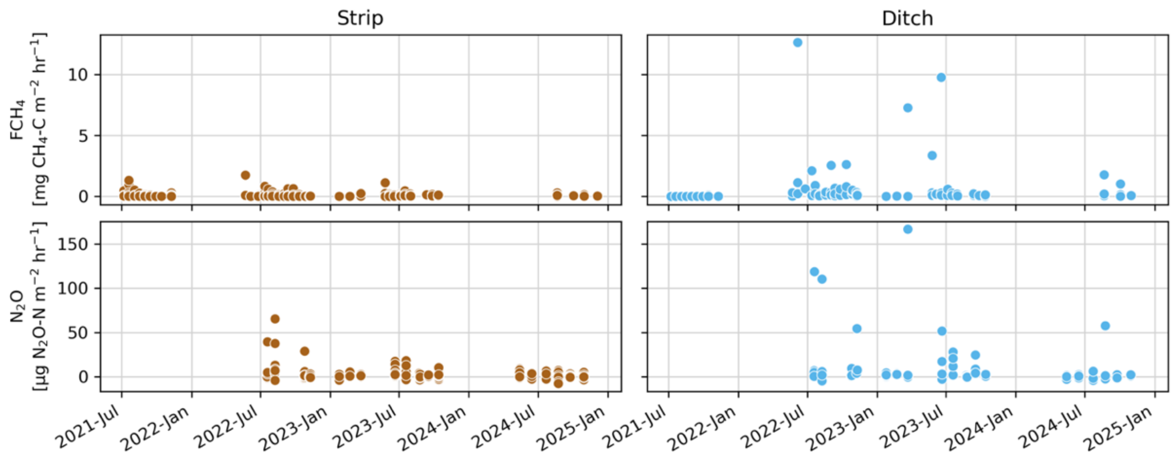

Figure S2.5. Timeseries of methane (FCH_4) and nitrous oxide fluxes (FN_2O) measured from strips (left column) and ditches (right column) by the manual chamber method at Naarasneva.

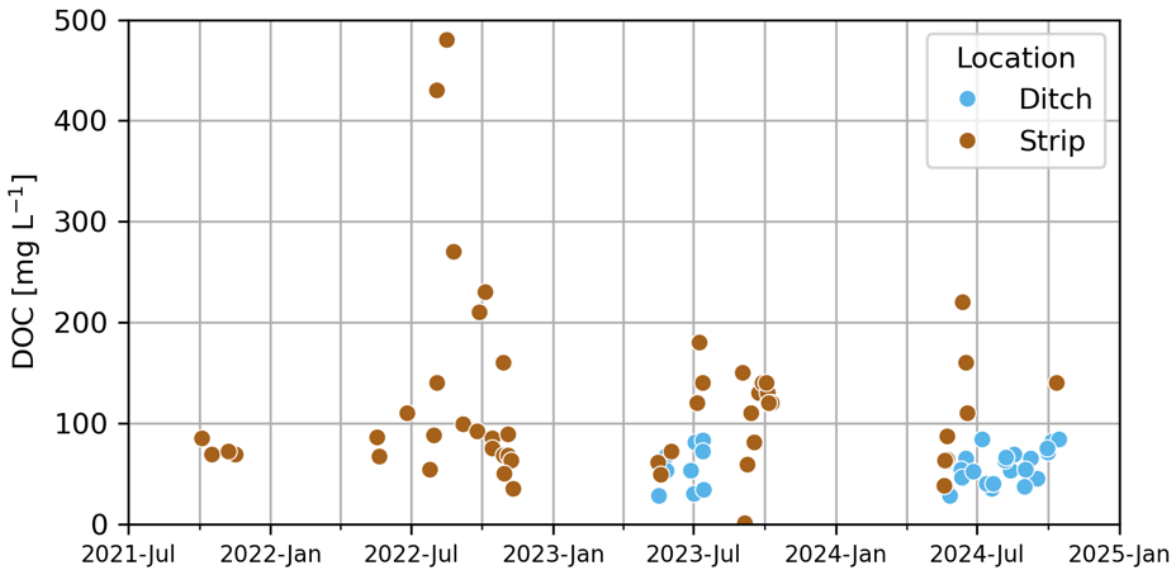

Figure S2.6. Timeseries of dissolved organic carbon (DOC) concentrations measured from samples taken at Naarasneva.
