# Supplementary material for: Afforestation‐Related Fertilisation Quickly Turns Barren Cutaway Peatland Into a Carbon Dioxide Sink
Source: Glob Chang Biol. 2025 Dec 17;31(12):e70644. doi: 10.1111/gcb.70644 (PMC12710595; doi:10.1111/gcb.70644)
Supplement: Supplementary file 3 — Supporting Information S3: gcb70644‐sup‐0003‐Supinfo3.pdf. [file GCB-31-e70644-s002.pdf]

Supporting information S3 to:

Afforestation-related fertilisation quickly turns barren cutaway peatland into a carbon dioxide sink

Alexander J.V. Buzacott[1], Kari Laasasenaho[2], Risto Lauhanen[2], Kari Minkkinen[3], Paavo Ojanen[3,4], Gopal Adhikari[3], Liisa Jokelainen[3], Lassi Päkkilä[5], Hannu Marttila[5], Annalea Lohila[1,6]

[1]Institute for Atmospheric and Earth System Research/Physics, University of Helsinki, Pietari Kalmin katu 5, Helsinki, 00560, Finland
[2]Seinäjoki University of Applied Sciences, P.O. Box 412 / Frami F, Kampusranta 11, FI-60101 Seinäjoki, Finland
[3]Department of Forest Sciences, University of Helsinki, Latokartanonkaari 9, 00790 Helsinki, Finland
[4]Natural Resources Institute Finland, Latokartanonkaari 9, 00790 Helsinki, Finland
[5]Water, Energy and Environmental Engineering Research Unit, Faculty of Technology, P.O. Box 4300, FI-90014 University of Oulu, Finland
[6]Climate System Research, Finnish Meteorological Institute, Erik Palménin aukio 1, Helsinki, 00560, Finland

Correspondence to: Alexander J.V. Buzacott (alexander.buzacott@helsinki.fi)

Contents:

- **Figure S3.1.** Distributions of methane (FCH_4) and nitrous oxide (FN_2O) fluxes measured using the chamber method. The left column shows the observed distributions, and the right column shows the distributions after transformation using the natural logarithm (ln).

- **Figure S3.2.** The relationships of ditch methane fluxes (FCH_4) and air temperature (TA) and strip FCH_4 and soil temperature (TS) at 5 cm. The relationships are shown at the raw level and daily median of the site level, as well as in their observed distribution (top row) and natural log (ln) transformed distribution (bottom row). The different colours of the raw measurements indicate the measurement plots north (N), east (E), south (S), west (W).

- **Figure S3.3.** The relationships of ditch nitrous oxide fluxes (FN_2O) and air temperature (TA) and strip FN_2O and soil temperature (TS) at 5 cm. The relationships are shown at the raw level and daily median of the site level, as well as in their observed distribution (top row) and natural log (ln) transformed distribution (bottom row). The different colours of the raw measurements indicate the measurement plots north (N), east (E), south (S), west (W).

- **Figure S3.4.** The relationships of strip methane fluxes (FCH_4) with water level (WL) and soil water content (SWC) measured at 10 cm. The relationships are shown at the raw level and daily median of the site level, as well as in their observed distribution (top row) and natural log (ln) transformed distribution (bottom row). The different colours of the raw measurements indicate the measurement plots north (N), east (E), south (S), west (W).

- **Figure S3.5.** The relationships of strip nitrous oxide fluxes (FN_2O) with water level (WL) and soil water content (SWC) measured at 10 cm. The relationships are shown at the raw level and daily median of the site level, as well as in their observed distribution (top row) and natural log (ln) transformed distribution (bottom row). The different colours of the raw measurements indicate the measurement plots north (N), east (E), south (S), west (W).

Before assessing relationships between fluxes and drivers, the fluxes of methane (FCH_4) and nitrous oxide (FN_2O) were normalised by log transformation (Figure S3.1, also shown in Figure 7a,b in the main article). The linear correlation of strip FCH_4 to soil temperature at 5 cm (TS) was poor (r=0.25, n=449), while the relationship of ditch FCH_4 and air temperature (TA) was better (r=0.41, n=93) (Figure S3.2). After taking the site-level median of daily emissions, the relationship between the strip FCH_4 and TS improved substantially (r=0.55, n=39) but only marginally for ditches (r=0.59, n=35). There was a poor relationship between the strip FN_2O and TS (r=0.25, n=176) and ditch FN_2O and TA (r=0.14, n=54) (Figure S3.3). The relationships improved marginally when using the site-level daily median of strip FN_2O (r=0.46, n=20) but not for ditches (r=0.08, n=19).

Weak relationships were found between the strip FCH_4 (r=−0.22, n=211) and FN_2O (r=−0.06, n=71) to plot water level (WL) (Figure S3.4, S3.5). At the median daily site-level, the relationship was poorer for strip FCH_4 (r=−0.06, n=34) and better for FN_2O (r=−0.60, n=16). Similarly poor results were found between strip FCH_4 (r=−0.11, n=399) and FN_2O (r=−0.26, n=176) and soil water content at 10 cm (SWC) measured by the EC tower (Figure S3.4, S3.5). The correlation strength did not improve at the median daily site-level for FCH_4 (r=−0.09, n=37) but did for FN_2O (r=−0.53, n=20). For both WL and SWC the relationships are unintuitive and suggest lower fluxes with decreasing WL and SWC. There were no improvements found using partial correlations to try and account for temperature effects in the relationship between fluxes and WL and SWC.

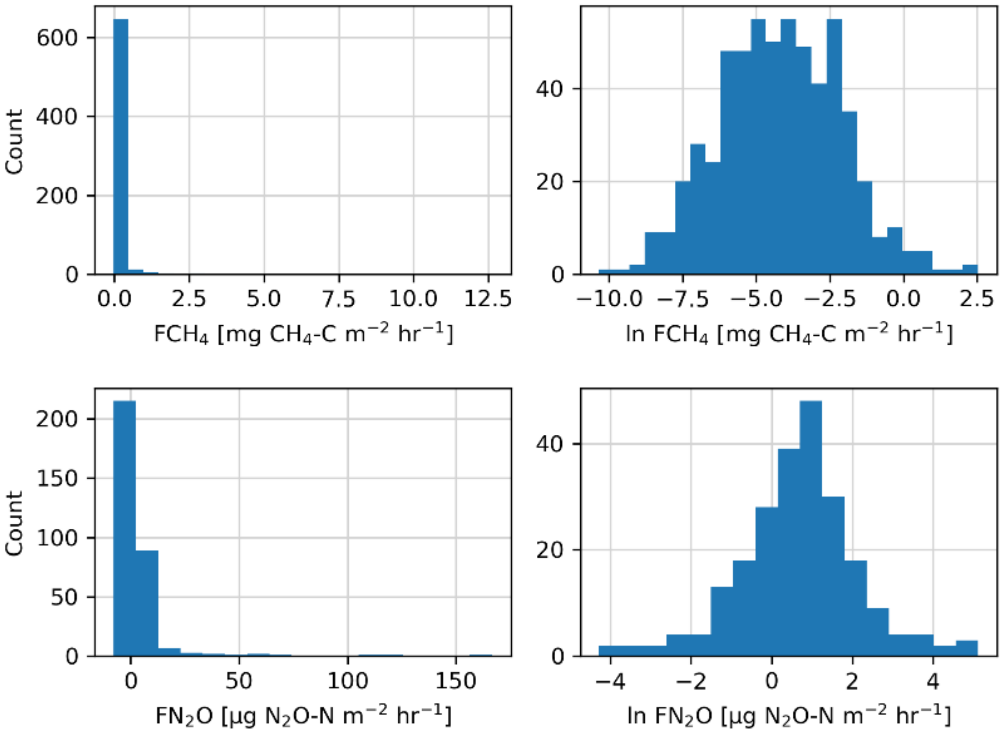

Figure S3.1. Distributions of methane (FCH_4) and nitrous oxide (FN_2O) fluxes measured using the chamber method. The left column shows the observed distributions, and the right column shows the distributions after transformation using the natural logarithm (ln).

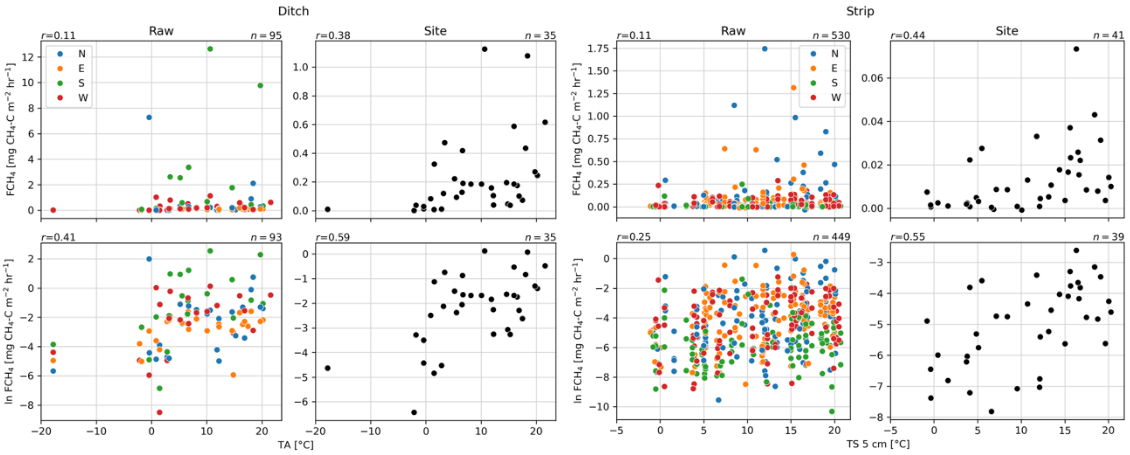

Figure S3.2. The relationships of ditch methane fluxes (FCH4) and air temperature (TA) and strip FCH4 and soil temperature (TS) at 5 cm. The relationships are shown at the raw level and daily median of the site level, as well as in their observed distribution (top row) and natural log (ln) transformed distribution (bottom row). The different colours of the raw measurements indicate the measurement plots north (N), east (E), south (S), west (W).

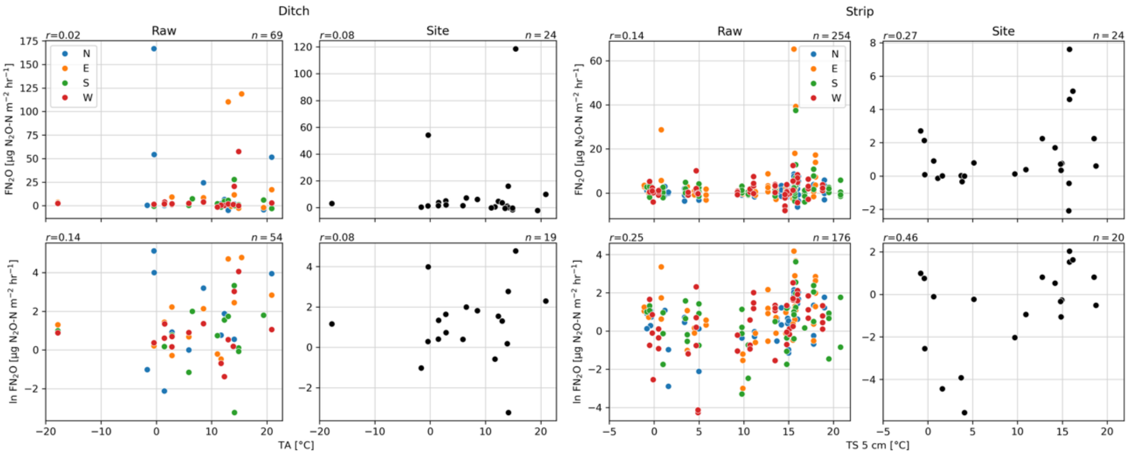

Figure S3.3. The relationships of ditch nitrous oxide fluxes (FN_2O) and air temperature (TA) and strip FN_2O and soil temperature (TS) at 5 cm. The relationships are shown at the raw level and daily median of the site level, as well as in their observed distribution (top row) and natural log (ln) transformed distribution (bottom row). The different colours of the raw measurements indicate the measurement plots north (N), east (E), south (S), west (W).

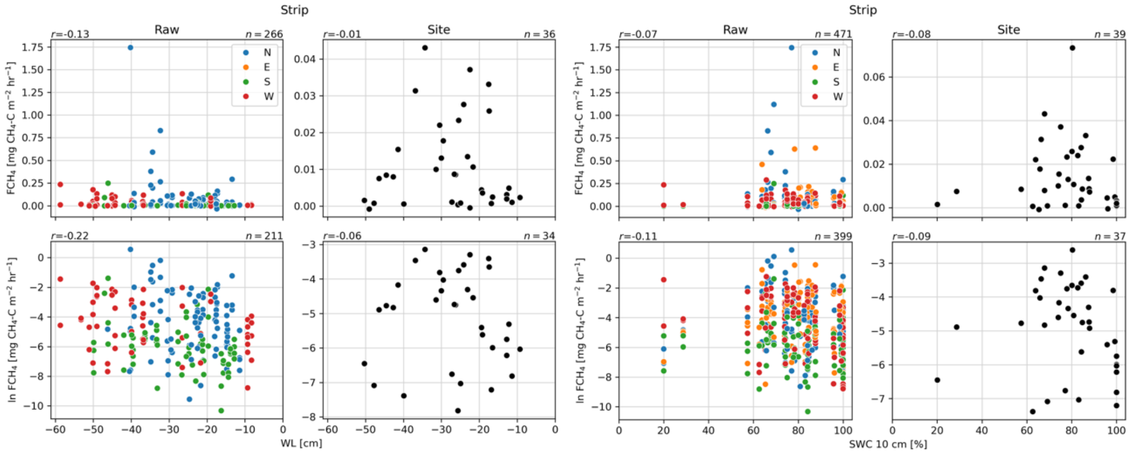

Figure S3.4. The relationships of strip methane fluxes (FCH_4) with water level (WL) and soil water content (SWC) measured at 10 cm. The relationships are shown at the raw level and daily median of the site level, as well as in their observed distribution (top row) and natural log (ln) transformed distribution (bottom row). The different colours of the raw measurements indicate the measurement plots north (N), east (E), south (S), west (W).

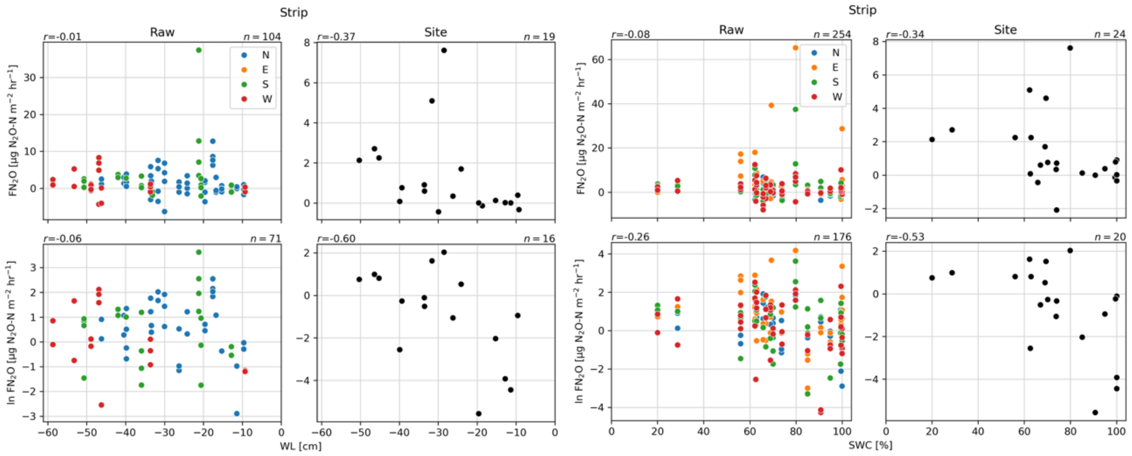

Figure S3.5. The relationships of strip nitrous oxide fluxes (FN_2O) with water level (WL) and soil water content (SWC) measured at 10 cm. The relationships are shown at the raw level and daily median of the site level, as well as in their observed distribution (top row) and natural log (ln) transformed distribution (bottom row). The different colours of the raw measurements indicate the measurement plots north (N), east (E), south (S), west (W).
